# The P-glycoprotein repertoire of the equine parasitic nematode *Parascaris univalens*

**DOI:** 10.1038/s41598-020-70529-6

**Published:** 2020-08-12

**Authors:** Alexander P. Gerhard, Jürgen Krücken, Emanuel Heitlinger, I. Jana I. Janssen, Marta Basiaga, Sławomir Kornaś, Céline Beier, Martin K. Nielsen, Richard E. Davis, Jianbin Wang, Georg von Samson-Himmelstjerna

**Affiliations:** 1 Institute for Parasitology and Tropical Veterinary Medicine, Freie Universität Berlin, Berlin, Germany; 2 Institute of Biology, Molecular Parasitology, Humboldt-Universität Zu Berlin, Berlin, Germany; 3 Leibniz Institute for Zoo and Wildlife Research, Research Group Ecology and Evolution of Parasite Host Interactions, Berlin, Germany; 4 Department of Zoology and Animal Welfare, University of Agriculture in Kraków, Kraków, Poland; 5 Maxwell H. Gluck Equine Research Center, University of Kentucky, Lexington, USA; 6 Department of Biochemistry and Molecular Genetics, RNA Bioscience Initiative, University of Colorado School of Medicine, Aurora, USA; 7 Department of Biochemistry and Cellular and Molecular Biology, University of Tennessee, Knoxville, TN USA

**Keywords:** Transcriptomics, Parasite genetics, Gene expression profiling, Sequence annotation, Genetics, Gene expression

## Abstract

P-glycoproteins (Pgp) have been proposed as contributors to the widespread macrocyclic lactone (ML) resistance in several nematode species including a major pathogen of foals, *Parascaris univalens*. Using new and available RNA-seq data, ten different genomic loci encoding Pgps were identified and characterized by transcriptome-guided RT-PCRs and Sanger sequencing. Phylogenetic analysis revealed an ascarid-specific Pgp lineage, Pgp-18, as well as two paralogues of Pgp-11 and Pgp-16. Comparative gene expression analyses in *P. univalens* and *Caenorhabditis elegans* show that the intestine is the major site of expression but individual gene expression patterns were not conserved between the two nematodes. In *P. univalens*, *Pun*Pgp-9, *Pun*Pgp-11.1 and *Pun*Pgp-16.2 consistently exhibited the highest expression level in two independent transcriptome data sets. Using RNA-Seq, no significant upregulation of any Pgp was detected following in vitro incubation of adult *P. univalens* with ivermectin suggesting that drug-induced upregulation is not the mechanism of Pgp-mediated ML resistance. Expression and functional analyses of *Pun*Pgp-2 and *Pun*Pgp-9 in *Saccharomyces cerevisiae* provide evidence for an interaction with ketoconazole and ivermectin, but not thiabendazole. Overall, this study established reliable reference gene models with significantly improved annotation for the *P. univalens* Pgp repertoire and provides a foundation for a better understanding of Pgp-mediated anthelmintic resistance.

## Introduction

Parasitic nematodes are important pathogens of livestock, companion animals and humans and the emergence and spread of anthelmintic resistance has compromised veterinary helminth control. In equines, *Parascaris univalens* poses a major threat particularly to juvenile horses^1,2^. At present, chemotherapeutic metaphylaxis and therapy remain the most effective and commonly used strategies for veterinary helminth control although widespread anthelmintic resistance to one or multiple drug classes compromise their success^3–5^. The frequent and unrestricted use of macrocyclic lactones (MLs) in domestic horses over decades has driven selection of resistant parasite populations^6^. In *Parascaris* sp., ML resistance was first reported in the early 2000s^7^ and since then has developed into a global challenge. Its presence has been demonstrated in several European countries^8–13^, North America^14–16^, as well as New Zealand^17^, Australia^18^ and more recently Ethiopia^19^ and Saudi-Arabia^20^. Similarly, the development and spread of ML resistance has been reported in many other parasitic nematodes, including *Teladorsagia circumcincta*^21^ and *Haemonchus contortus* in sheep^22^, *Dirofilaria immitis*^23^ in dogs and even in human filarial nematodes such as *Onchocerca volvulus*^24^.

Prior to genome-wide approaches, P-glycoproteins (Pgp) were already presumed to be important contributors to ML resistance in a number of parasitic nematodes^25^. Nonetheless, deciphering the mechanisms of ML resistance is challenging, partly due to the multi-genic nature of the resistance traits^26,27^. Furthermore, inconsistency between different species or populations in different studies reveals the high complexity of this problem. For instance, multigenicity has become apparent in light of recent genome-wide studies^28–30^, which also substantiate evidence for Pgp as candidate genes. Choi et al. showed in a backcross experiment conducted in *T. circumcincta* that ABC-transporters were among the genes that had undergone selection in response to treatments with ivermectin (IVM), a commonly used ML. In this study, *Tci*-Pgp-9 exhibited higher copy numbers in the genome, four strongly selected single nucleotide polymorphisms (SNPs) and a higher number of transcripts in RNA-seq in the resistant backcrossed isolate^29^. In contrast, genome-wide association studies in *H. contortus* are conflicting and either point towards a single major resistance locus, which does not directly correspond to any previous candidate resistance gene^31,32^ or multiple selected loci including Pgp^30^.

P-glycoproteins belong to the superfamily of ATP binding cassette (ABC) transporters and form the ABCB subfamily^33^. The substrates of these xenobiotic transporters are usually neutral or lipophilic^34,35^ and they exhibit a broad substrate range^36^. Their versatility as transmembrane transporters is most likely the reason for their evolutionary success — Pgps can be found in almost all eukaryotes and seem to have experienced an expansion through duplication events in several species giving rise to different Pgp lineages^37^. This evolutionary diversification has been particularly strong in nematodes^38^: while mammals only possess one or two different Pgp genes^37^, nematodes typically have a much larger and more diverse repertoire, for example 15 Pgps in *Caenorhabditis elegans*^39^, including a pseudogene^40^. Pgp open reading frames (ORFs) are comparatively large (usually 3,800–4,000 bp) and translate into proteins of about 170 kDa with a central pore formed by two transmembrane domains each containing six transmembrane helices followed by an ATP binding site^41^.

Although genome and transcriptomes of parasitic nematodes provide a crucial resource for anthelmintic resistance research, to date in silico prediction of transcripts from draft genome and limited transcriptomes is error prone and often leads to incomplete gene models for many, in particular larger genes. In the case of the diverse Pgp gene family, this has been a particular challenge as many studies (e.g. Janssen et al., 2013 and Jesudoss et al., 2019 on *Parascaris* sp.) have focused only on the few validated parasite Pgp genes, resulting in a research bias towards these genes.

The mechanism of anthelmintic drug resistance due to Pgps, has been linked to overexpression of individual Pgps in several parasitic nematode species, i.e. of Pgp-2 and Pgp-9 in *H. contortus*^42^, Pgp-9 in *T. circumcincta*^43^ as well as of Pgp-11 in *P. univalens*^44^. In line with findings for other nematode Pgps such as for *Tci*Pgp-9 from candidate gene and whole-genome studies^29,45^ as well as early studies on a *H. contortus* Pgp^46^, three SNPs identified in a *Parascaris* sp. Pgp-11 orthologue were strongly increased in field isolates of a resistant population compared to susceptible populations^44^. This suggests that both overexpression and drug target site mutations of Pgps may lead to a reduced IVM susceptibility. At a functional level, MLs have been shown to be substrates of Pgps. In this regard, avermectins such as IVM appear to be better substrates than milbemycins such as moxidectin^47–49^, most likely due to the lack of a sugar moiety of the latter^50^. Furthermore, the deletion of individual Pgps in *C. elegans* resulted in a modest increase of IVM susceptibility^51^, which appeared to vary slightly between individual Pgps. From the Pgp orthologues previously linked to ML and specifically IVM resistance through expression changes or signatures of selection, functional evidence for an interaction with MLs of *P. univalens* Pgp has only been provided for a Pgp-11 orthologue with IVM^52^ but not for orthologues of Pgp-2 and Pgp-9. In other nematodes, the Pgp-11 orthologue of *D. immitis*^53^ and the Pgp-2 and Pgp-9 orthologues of *H. contortus*^54,55^ have been shown to interact with MLs using the cell line LLC-PK1. Likewise, the Pgp-9 orthologue which has been linked most often to ML resistance in different parasitic nematodes at the epidemiological level, has also been shown to interact with MLs from *Cylicocyclus elongatus* using the *Saccharomyces cerevisiae* AD1-7 yeast strain^47^. Transgenic Pgp expression in this yeast strain lacking 7 endogenous ABC-transporters (AD1-7) has been established as an easy and cost-effective experimental approach to examine interaction directly with the antimycotic and anthelmintic thiabendazole (TBZ) and indirectly with MLs through co-incubation with the antimycotic and known Pgp-substrate ketoconazole (KCON).

In light of the aforementioned candidate gene and genome-wide studies and with respect to the high number of Pgp genes in nematodes, evidence is accumulating that only some Pgps are directly involved in ML resistance. To gain a better understanding of the anthelmintic resistance mechanisms, it is essential to know the whole inventory of Pgps in resistant nematode species. On these grounds we have identified and characterized the whole Pgp gene family of the frequently ML resistant ascarid species *P. univalen*s at the genomic and transcriptomic level. Following this comprehensive approach, the putative candidate Pgp orthologues *Pun*Pgp-9 and *Pun*Pgp-2 were characterized regarding their interaction with two important anthelmintic drug classes, macrocyclic lactones (IVM) and benzimidazoles (TBZ).

## Results

### Comprehensive annotation of full length P-glycoproteins

As Pgp annotation based on the *P. univalens* transcriptome assembly WormBase ParaSite version WPBS14 was incomplete for most Pgps and resulted in discontinuous ORFs, a transcriptome-guided RT-PCR approach was used allowing amplification and sequencing of all identified Pgps, revealing a total repertoire of ten Pgps in *P. univalens*.

Overall, annotation was considerably improved compared to the automatic annotation by Wang et al.^56^ automatic annotations WormBase ParaSite version WPBS14 for all Pgps with several novel exons added to the gene models (Supplementary Fig. S1). Specifically, 5′ and 3′ coding exons were missing for several Pgps (Supplementary Fig. S1), a large number of insertions, mismatches and deletions was detected disrupting the originally predicted ORFs (Supplementary Table S1) and in case of *Pun*Pgp-3 and *Pun*Pgp-12, annotation was severely fragmented and incomplete (Fig. S1b,g). For several splice junctions of previously annotated (in the WormBase ParaSite version WPBS14) and correctly identified exons, accuracy was improved through elimination of a deviation of a few base pairs. The updated Pgp annotation was integrated into the whole genome annotation in gff3 format and uploaded on WormBase ParaSite (parasite.wormbase.org).

Heat maps of splice events of Splign-analysed cDNA sequences were mostly consistent between replicates and reflected by another approach calculated transcripts per kilobase million (TPM) expression levels (in heatmaps as fragments per kilobase of exon per million [FPKM]) with highest expression in the intestine for most transcripts. However, in several cases varying exon support depending on tissues was detected, e.g. E28 (3′ exon) in *Pun*Pgp-2 is found only in the testis (Supplementary Fig. S1a) and E1 (5′ exon) in *Pun*Pgp-9 is not detected in testis or carcass tissue samples (Supplementary Fig. S1c).

The only experimentally identified alternative splice variant was found in *Pun*Pgp-18 for E13/14 and named *Pun*Pgp-18A and *Pun*Pgp-18B. Both variants were also found in both transcriptome data sets (Supplementary Fig. S1i) including the RNA-Seq experiment where worms where incubated with IVM, henceforth addressed as the IVM transcriptome (“Biological material, genome and transcriptome resources”), which had high junction support for variant A in all samples as well as weak support for variant B in the control samples NTC3 and NTC7 (data not shown).

### Phylogenetic relationship of *Parascaris univalens* and other nematode P-glycoproteins

**Figure 1:**
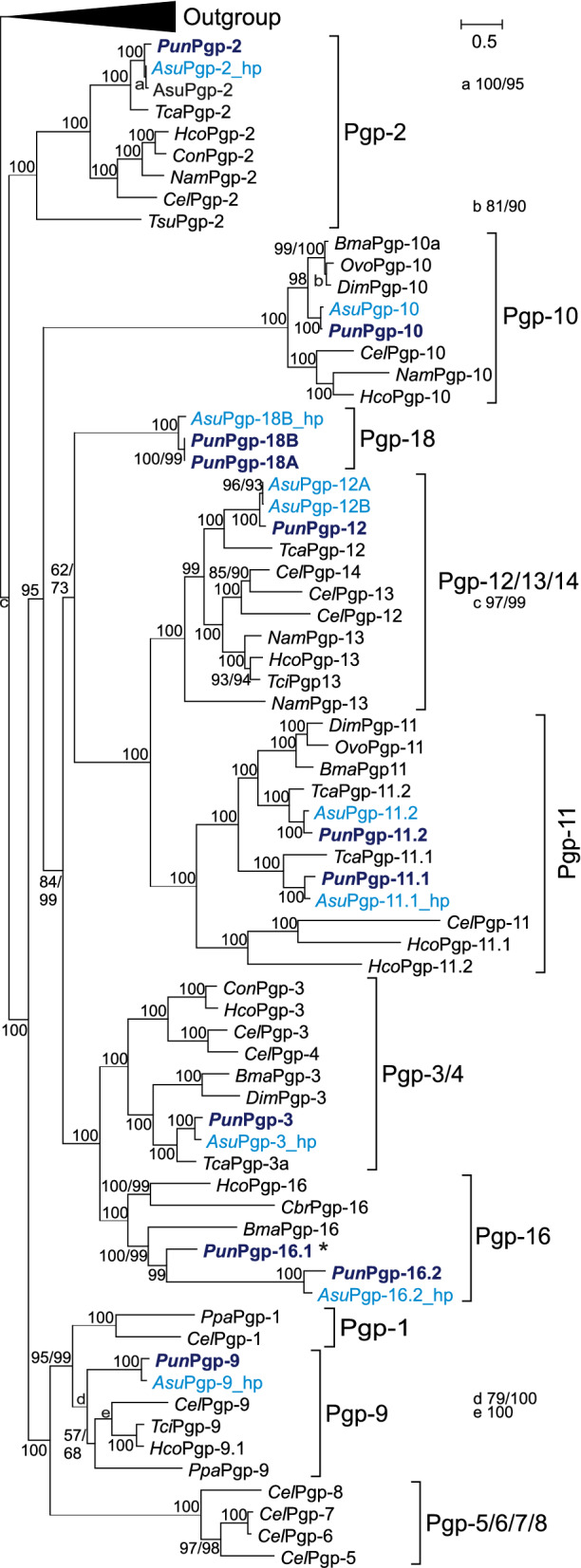
Phylogenetic analysis of *Parascaris univalens* and nematode Pgp. The consensus tree was calculated from Pgp protein sequences using RaxML and LG + F + G model allowing nearest neighbour interchange (NNI) and subtree pruning and regraftment (SPR) with 1,000 bootstrap replicates (first value). Thereafter, this tree was used to restrict tree topology in RaxML additional branch support values were calculated using the Shimodaira-Hasegawa (SH) approximate likelihood ratio test (second value). Nodes with only one value had the same support value calculated with both methods. A collapsed representative outgroup of *Drosophila melanogaster*, *Mytilus* spp., *Mus musculus*, *Pediculus humanus corporis* and *Homo sapiens* Pgps was used for rooting. *Parascaris univalens* Pgps are indicated in dark blue. *Ascaris suum* Pgps (light blue) from the sequence data of the most recent transcriptome assembly (GCA_000187025.3) are shown here as *Asu*Pgp-X_hp (hypothetical). *Asu*Pgp-16.1 was identified in the transcriptome but omitted in the analysis because of fragmented and incomplete sequence data and to indicate its presence *Pun*Pgp-16.1 is highlighted with an *. The scale bar represents the indicated number of substitutions per site. Accession numbers are given in Supplementary Table S1 as well as annotated protein names (changed in this figure according to the result of the phylogenetic analysis). Pgp: P-glycoprotein.

Phylogenetic analysis using complete protein sequences (Fig. 1) revealed that all outgroup sequences formed a single cluster, while nematode Pgps were separated into several strongly supported clusters containing *P. univalens* Pgps as well as their *C. elegans* orthologues. If applicable due to a very limited number of published full-length nematode Pgps, subclusters within a group of Pgp resembled general nematode phylogeny^57^ with separate ascarid, rhabditid and trichocephalid Pgp subclusters. Pgps of *P. univalens* and *A. suum* were named in accordance with CGC standards as orthologues of *C. elegans* as *Pun*Pgp-2, -3, -9, -10, -12, -11.1, -11.2, 16.1, and -16.2 and for hypothetical (manually corrected using the *P. univalens* orthologue full length ORF sequence) *A. suum* sequences *Asu*Pgp-X_hp. One Pgp lineage without a currently known orthologue in *C. elegans* or any parasitic nematode was identified in both *A. suum* and *P. univalens* and named Pgp-18. Several published reference Pgps were also renamed according to the result of the phylogenetic analysis. Remarkably, *P. univalens* appears to have two paralogues of Pgp-11 and Pgp-16, which were named Pgp-X.1 (previously published^44^) or -X.2, accordingly. Both *Pun*Pgp-11 paralogues possess an orthologue in *Toxocara canis* and *A. suum*. Here, *Pun*Pgp-11.2 shows stronger relatedness with filarial nematode Pgp-11 than *Pun*Pgp-11.1 for which no orthologue was identified in filariae. In contrast, *Pun*Pgp-16 paralogues bear stronger resemblance to each other than to the few other published Pgp-16 sequences of *Caenorhabditis briggsae* and *Brugia malayi,* but both paralogues have orthologues in *A. suum* as well. *Asu*Pgp-16.1 could not be assembled from the available contig sequences and was hence omitted from the phylogenetic analysis. With regard to their genomic positions, the Pgp-11 paralogues are separated and appear on different genomic scaffolds, NINM01000018.1 and NINM01000098.1 (Supplementary Fig. S1e,f), while *Pun*Pgp-16.1 and -16.2 are localized in close proximity (6,651 bp intergenic region) in a head to tail (Supplementary Fig. S1H) orientation on the genomic scaffold NINM01000014.1 with genomic coordinates 2,435,368:2,453,320 and 2,459,971:2,480,921, respectively.

All *P. univalens* Pgps have orthologues in *A. suum* and vice versa. Inspection of the alignment of complete translated cDNA sequences revealed large gaps and incongruity in regions localising in both TMDs of the Pgp. Despite that, a maximum likelihood tree (data not shown) calculated from a GBLOCKS edited alignment excluding sequence blocks with high divergence and ambiguous alignments, did not improve branch support values and was almost identical to the full length tree except for a shift in the position of the Pgp-10 cluster, which appeared in a sister position to the Pgp-2 cluster in the GBLOCKS edited tree in contrast to the tree calculated from the complete alignment (Fig. 1).

### Expression profiles of *Parascaris univalens* and *Caenorhabditis elegan*s P-glycoproteins

**Figure 2:**
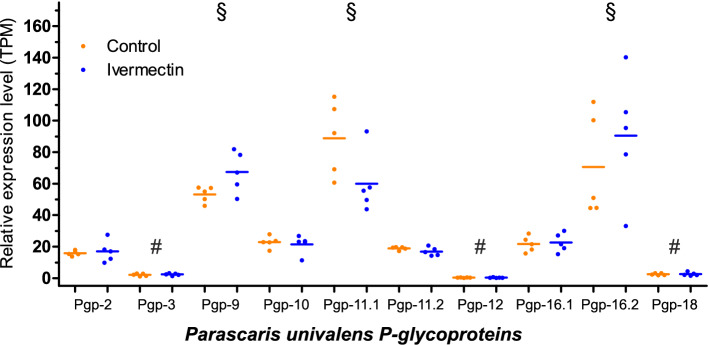
Relative expression levels of ivermectin and control incubated adult *Parascaris univalens*. Relative expression levels of *Parascaris univalens* adult females incubated with 10^–9^ M ivermectin (blue) or a DMSO control (orange) for twelve hours with 5 worms for each condition. Relative expression levels were derived from RNA-Seq raw read libraries. Reads were mapped onto the augmented (with *P. univalens* Pgp cDNA sequences) *P. univalens* genome (genome assembly ASM225920v1, version WBPS14) using STAR and featureCounts and normalised as transcripts per million (TPM), then each replicate was visualized per Pgp using GraphPad Prism v. 8.3.3 (GraphPad Software, San Diego, California USA, https://www.graphpad.com). No significant upregulation of any Pgps was found following the incubation of worms with ivermectin compared to the DMSO control groups (Kruskal–Wallis test, Dunn’s post-hoc test, *p* > 0.05) but constitutive expression levels of *Pun*Pgp-11.1, 16.2 and -9 (§) were significantly higher compared to *Pun*Pgp-3, -12 and -18 (#) (Kruskal–Wallis test, Dunn’s post-hoc test, p < 0.05) in both the control and the ivermectin incubated group. Pgp: P-glycoprotein.

IVM incubation of *P. univalens* did not result in a significantly changed expression level in terms of TPM of any of the Pgps compared to the DMSO incubated control group (Kruskal–Wallis, Dunn’s post-hoc test, p > 0.05) (Fig. 2). However, base expression levels within both the control group and the IVM incubated group were significantly higher for *Pun*Pgp-11.1, -16.2 and -9 compared to *Pun*Pgp-3, -12 and -18 (Kruskal–Wallis test, Dunn’s post-hoc test, p < 0.05) (Fig. 2). In between the high (TPM > 40) and low expression Pgps (TPM < 5), *Pun*Pgp-2, -10, -11.2 and -16.1 formed a group with intermediate TPM expression (Fig. 2).

**Figure 3:**
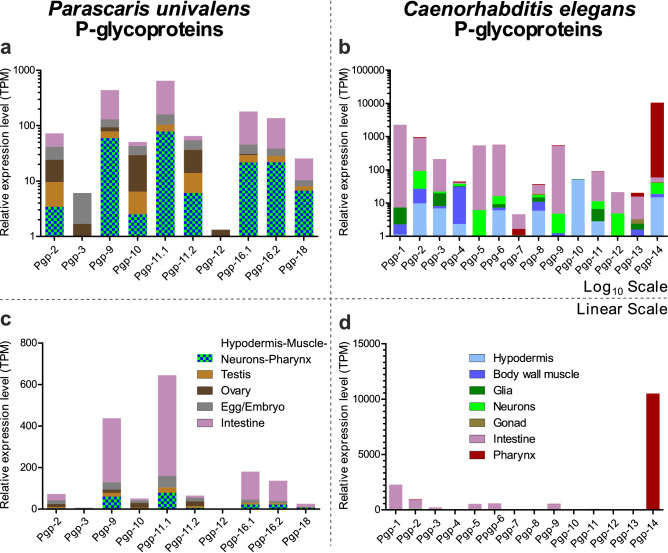
Relative tissue expression levels of *Parascaris univalens* and *Caenorhabditis elegans* P-glycoproteins. Tissue expression levels of *Parascaris univalens* worms were calculated based on transcriptome raw reads data sets (Geo Accession GSE99524, samples GSM2645460 to GSM2645464) mapped onto the augmented (with *P. univalens* Pgp cDNA sequences) *P. univalens* genome (WormBase ParaSite genome assembly ASM225920v1, version WBPS14) using STAR. Expression levels were normalised as transcripts per million (TPM) from raw read counts obtained with featureCounts. *Caenorhabditis elegans* tissue expression levels (TPM) were obtained from a single cell transcriptome (Geo accession number: GSE98561) and calculated with R^87^ package monocle3^88–90^. Then expression levels of *P. univalens* (**a**) and (**b**) and *C. elegans* (**b**) and (**d**) were visualized on a log_10_ scale (**a**) and (**b**) and a linear scale (**c**) and (**d**). The different tissues were color-coded as followed. Digestive tract: *P. univalens* and *C. elegans* intestine (purple), *C. elegans* pharynx (red); Hypodermis/Cuticle/Neuron: *C. elegans* hypodermis (light blue), body wall muscle (dark blue), glia (dark green) and neurons (light green), *P. univalens* hypodermis-neuronal-muscle tissue (blue-green); Reproductive tissue *C. elegans* gonads (brown); *P. univalens* ovary (dark brown), testis (light brown). Visualisation was done using GraphPad Prism v. 8.3.3 (GraphPad Software, San Diego, California USA, https://www.graphpad.com). *Pgp* P-glycoprotein.

In a *P. univalens* transcriptome data set with tissue specific samples from a population of worms naïve to anthelmintics, henceforth addressed as the tissue specific transcriptome (“Biological Material, genome and transcriptome resources”), both replicates exhibited almost identical TPM expression levels for all Pgps (Supplementary Fig. S2). As sample size (n = 2) was too small, no statistical analysis was conducted. However, expression levels and patterns varied considerably between different tissues. To improve information depth in the graphs, expression levels were visualised on a log_10_ scale for *P. univalens* (Fig. 3a) and as a comparison for *C. elegans* (Fig. 3b) as well as on a linear scale for both nematodes (*P. univalens* Fig. 3c and *C. elegans* Fig. 3d). Similar to the IVM-transcriptome, *Pun*Pgp-9, -11.1 and the two *Pun*Pgp-16 paralogues were the most strongly expressed Pgps in the tissue specific transcriptome (Fig. 3c). The intestine exhibited by far the strongest overall expression and in particular of those same four aforementioned Pgps (Fig. 3c). This strong intestinal Pgp expression was also found in *C. elegans* where expression analysis was based on a single cell transcriptome data set^58^, although here the extraordinarily strong pharyngeal expression of *Cel*Pgp-14 (missing in *P. univalens*) was most striking (Fig. 3d). Second highest Pgp expression in *P. univalens* was found in the carcass tissue (hypodermis, muscle, neuron and pharynx) (Fig. 3a). In contrast, in *C. elegans* neuronal, body wall muscle and hypodermis expression was observed for almost all Pgps although at very low levels (Fig. 3b). In the gonadal tissues only moderate to low Pgp expression was found in *P. univalens* (Fig. 3a) with differences between the sexes, i.e. strong expression of *Pun*Pgp-10 and -11.2 in the ovaries which was low in the testis and in any of the other tissues (Fig. 3c). In contrast in the testis, expression of *Pun*Pgp-9 and -11.1 was strongest, which in turn was very low in the ovary (Supplementary Fig. S2). Pgp expression in *C. elegans* was almost completely absent in the gonads, except for very low expression of *Cel*Pgp-2, -8 and -14 (Fig. 3b) and low in glia and neurons (Fig. 3b). Overall, *Pun*Pgp-9 and -11.1 and the Pgp-16 paralogues were the most strongly expressed in almost all tissues, except for the ovary (Supplementary Fig. S2a).

### Functional analysis of *Pun*Pgp-2 and *Pun*Pgp-9 in a yeast growth assay

The *Saccharomyces cerevisiae* AD1234567 strain was successfully transformed with the genes *Pun*Pgp-2, *Pun*Pgp-9 and lacZ in the pYes2 vector and expressed under the control of the *gal-1* promoter. For each gene, transcription was validated in at least one clone by RT-PCR (Supplementary Fig. S3a for lacZ and b for *Pun*Pgp-2/-9). For each clone, clone 1 was used for all analyses. The generated strains are henceforth referred to as AD1-7Pgp-9, AD1-7Pgp-2 and AD1-7lacZ. The AD1-7 strain lacks seven endogenous xenobiotic ABC-transporters and hence exhibits an increased susceptibility to antimycotic ABC-transporter substrates such as KCON. Therefore, transgenic Pgp overexpression is expected to lead to measurable shifts in susceptibility.

**Figure 4:**
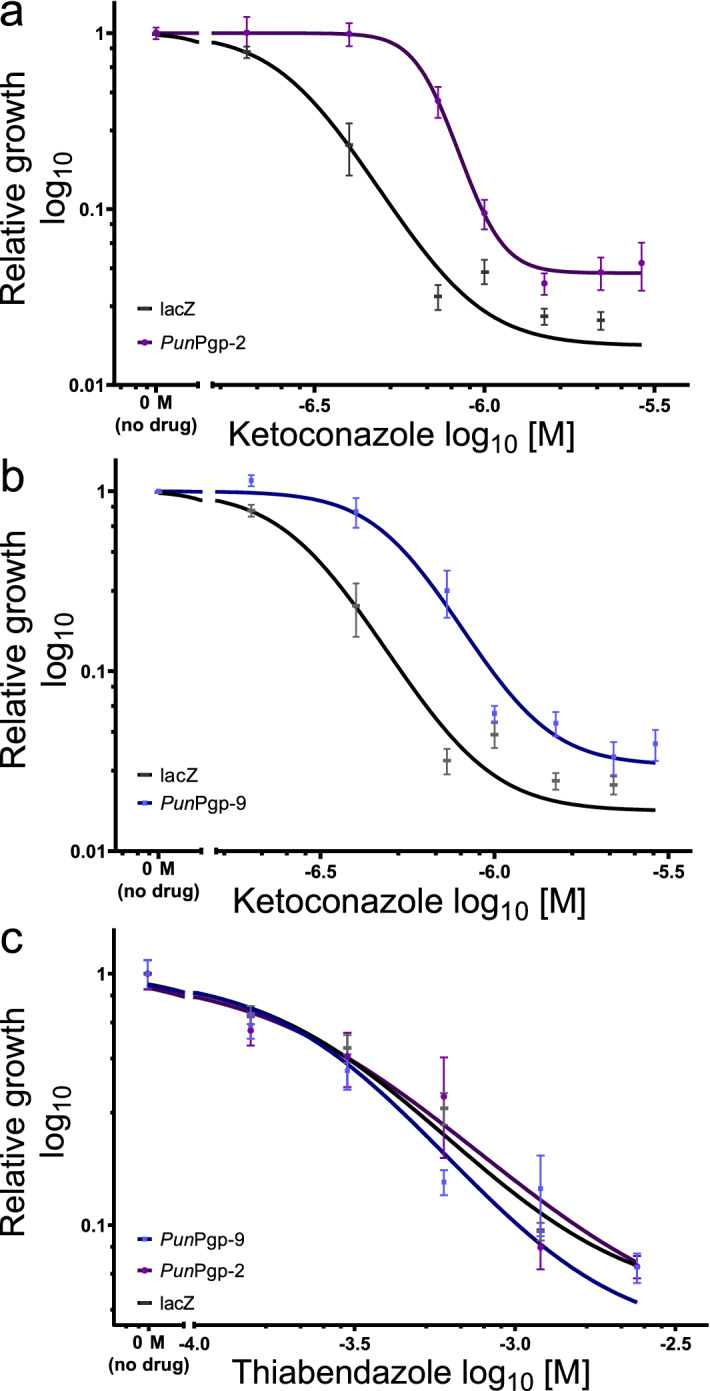
Susceptibility of AD1-7 expressing *Pun*Pgp-2 and *Pun*Pgp-9 to ketoconazole and thiabendazole. Susceptibility to ketoconazole was determined as relative growth of transgenic *Saccharomyces cerevisiae* AD1-7 strain in the presence of a dilution series of ketoconazole (final concentration of 1% DMSO) or thiabendazole (dissolved in water) with log_10_ transformation of concentrations, setting the negative no drug control to 10^–7^ M (ketoconazole) and 10^–4.15^ M (thiabendazole) but visualising it as “0 M (no drug)” separated by a break in the x-axis. Yeast were grown in 48-well plates with 12 replicates, four each on three separate days, per strain and concentration for 48 h and measuring OD_600_ every 10 min. From blanked reads (to wells containing only medium but no yeast), the area under the curve was calculated and then normalised to the no-drug control for the same strain on the same day. Then, four-parameter non-linear regression models were calculated and compared using the extrasum-of-squares F test with Holm’s adjustment. Relative growth curves for AD1-7Pgp-2 (**a**) (purple circles) and AD1-7Pgp-9 (**b**) (blue squares) exhibited significantly increased EC_50_s for ketoconazole (p = 0.0002) but not to thiabendazole (**c**) (p = 0.7897 and AD1-7Pgp-9 p = 0.498) compared to AD1-7lacZ strain (black rectangles). Calculation and visualisation was performed using GraphPad Prism v. 8.3.3 (GraphPad Software, San Diego, California USA, https://www.graphpad.com). Pgp: P-glycoprotein.

To confirm that the identified Pgps have a function in xenobiotic transport two Pgp orthologues implicated with ML resistance in other nematodes but which have not been studied in *P. univalens* were chosen, i.e. *Pun*Pgp-2 and *Pun*Pgp-9. Ketoconazole inhibited yeast growth in a concentration dependant manner with an EC_50_ of 0.28 µM KCON in the AD1-7lacZ strain (Table 1). Expression of both *Pun*Pgp-2 and *Pun*Pgp-9 resulted in a reduction of KCON susceptibility visible in a marked curve shift to the right (Fig. 4 a and b) with significantly increased EC_50_ values (p = 0.0002) of 0.58 µM KCON (2.46 fold increase) and 0.44 µM KCON (1.86 fold increase) for the two strains, respectively (Table 1). Thiabendazole also inhibited the growth of yeasts with an EC_50_ of 0.16 mM TBZ in the AD1-7lacZ strain (Table 1). In contrast to KCON, expression of both Pgp resulted in only minor changes in the concentration–response-curves (Fig. 1c) and not in a significant change in TBZ susceptibility with EC_50_ values of 0.15 mM TBZ and 0.24 mM TBZ for AD1-7Pgp-2 and AD1-7Pgp-9, respectively (Table 1).

**Table 1:** EC_50_s of transgenic AD1-7 *Saccharomyces cerevisiae* relative growth in the presence of ketoconazole or thiabendazole.

Strain	EC_50_ ketoconazole (µM)	EC_50_ 95% CI (µM)	Fold change^a^	R^2^	p-value^b^
AD1-7lacZ	0.28	0.25–0.31	–	0.89	
AD1-7Pgp-2	0.69	0.58–0.82	2.46	0.57	0.0002
AD1-7Pgp-9	0.52	0.44–0.62	1.86	0.69	0.0002

Strain	EC_50_ thiabendazole (mM)	EC_50_ 95% CI (mM)	Fold change^a^	R^2^	p-value^b^
AD1-7lacZ	0.16	0.13–0.48	–	0.47	
AD1-7Pgp-2	0.15	0.101–0.39	0.94	0.65	0.79
AD1-7Pgp-9	0.24	0.04–0.94	1.47	0.54	0.498

*Pgp* P-glycoprotein; *EC*_*50*_ half maximal effective concentration calculated from four parameter non-linear regression model; *CI* confidence interval.

^a^EC_50_ fold change compared to the lacZ expressing control strain.

^b^Extra sum-of-squares F test with Holm’s correction for multiple testing.

**Figure 5:**
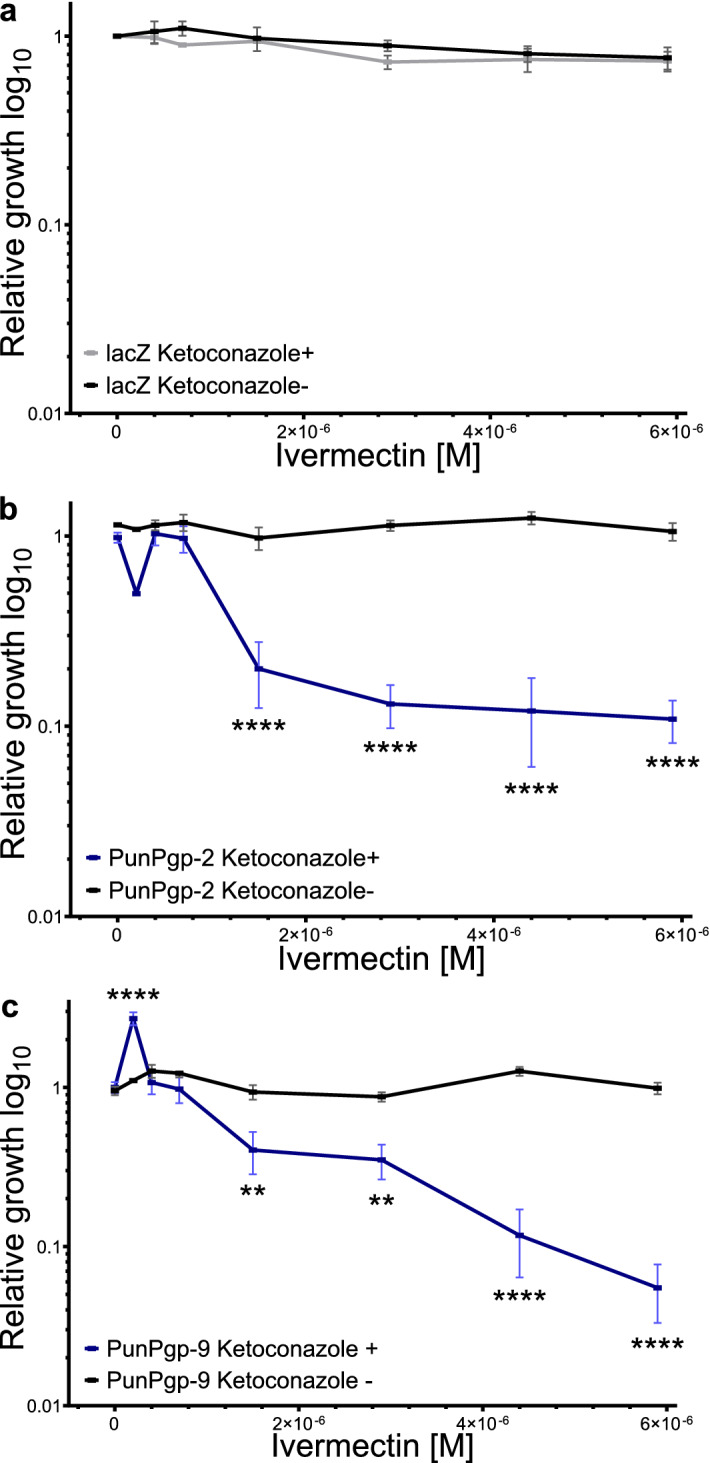
Relative transgenic yeast growth inhibition by ivermectin in presence or absence of ketoconazole. Relative growth was determined by growing transgenic *Saccharomyces cerevisiae* AD1-7 strain expressing lacZ (**a**) (grey/black) *Pun*Pgp-2 (**b**) (purple/black) or *Pun*Pgp-9 (**c**) (blue/black) in the presence of a dilution series of ivermectin either with or without (black) a maximum tolerated ketoconazole concentration (0.73 µM in AD1-7Pgp-2 and Pgp-9 and 0.2 µM in AD1-7lacZ) on a 48 well plate for 48 h, measuring OD_600_ every 10 min for 12 replicates on three separate days. Then, following blanking to empty wells, the area under the curve was calculated which was normalised to the growth of the negative no drug control of the same day. For each strain, relative growth was compared at each concentration in the presence or absence of ketoconazole using a two-way ANOVA followed by a Dunn’s post-hoc test. Calculations were done using R version 3.6.2^87^ and package dunn.test^93^. **p < 0.01, ****p < 0.0001. *Pgp* P-glycoprotein.

Ivermectin did not inhibit yeast growth in the AD1-7Pgp-2 and AD1-7lacZ strain (p = 0.796 and 0.168, one-way ANOVA). In the AD1-7Pgp-9 strain relative growth varied significantly between concentrations (p < 0.0001, one-way ANOVA) but was not significantly inhibited at any concentration (adjusted p > 0.05, Tukey’s honestly significant difference (HSD) post-hoc test). However, relative growth was significantly elevated at 0.4 µM IVM compared to the control strain (adjusted p < 0.0001, p > 0.05 Tukey’s HSD post-hoc test with Holm’s correction for multiple testing). Since IVM did not directly inhibit yeast growth (Fig. 5 black curves), an indirect assay was used to detect potential interaction with Pgps. A low KCON concentration, that only marginally reduced growth of the particular yeast strains, was used and combined with increasing IVM concentrations. A concentration of 0.2 µM KCON was used for AD1-7lacZ strain, while 0.73 µM KCON was chosen for both Pgp expressing strains (Fig. 4 a and b). However, in the presence of KCON, increasing IVM concentrations resulted in the inhibition of yeast growth in the AD1-7Pgp-2 and AD1-7Pgp-9 strains (Fig. 5b,c) but not in the AD1-7lacZ strain (Fig. 5a). For both AD1-7Pgp-2 and AD1-7Pgp-9, a significant growth inhibition was measured at concentrations higher than 0.73 µM IVM in the presence of KCON (all adjusted p < 0.05 two-way ANOVA and a Dunn’s post hoc test with Holm’s p-value adjustment). In contrast, no growth inhibition was observed in AD1-7lacZ in the presence of IVM (Fig. 5a) (all adjusted p > 0.05 two-way ANOVA and a Dunn’s post hoc test with Holm’s p-value adjustment).

## Discussion

Pgps are central components of the xenobiotic detoxification machinery in nematodes and the evolutionary success of this gene family in nematodes is reflected by its high diversity. For the first time the present study has identified the complete, manually curated Pgp repertoire of a clade III nematode with reliable gene models. The *P. univalens* repertoire comprising 10 Pgps resembles the *A. suum* repertoire and is similar in size to that of other parasitic nematodes such as 10 in *H. contortus*^59^ and 8 in *B. malayi*^60^. In addition, for the first time evidence for an interaction between *Pun*Pgp-2 and *Pun*Pgp-9 with IVM was provided, whereas TBZ did not appear to be a substrate of either of the two Pgps.

In ascarids, the evolution of this gene family remains a dynamic process in recent evolutionary history with gene duplications and subsequent divergence in Pgp-11 and Pgp-16 and a novel ascarid-exclusive lineage, Pgp-18. The closer relatedness between nematode Pgp lineages compared to the outgroup containing mammalian, mollusc and arthropod Pgps confirms the expansion of Pgps in an early nematode ancestor, but several nematode groups have lost some ancient or evolved new Pgp lineages. Regarding the novel Pgp-18 lineage, the lineage branches deep in the Pgp family but support for the particular position of this cluster appears to be comparatively weak in our phylogenetic analysis (62/73). No matter if it should be considered as a sister group to Pgp-11/12/13/14 or alternatively to Pgp-3/4/16, the position suggests that this family has split from the other clusters early in the evolution of ascarids. Comparably, the duplications in *Pun*Pgp-11 and *Pun*Pgp-16 have probably occurred after ascarid secession from filarial worms but before the differentiation into extant ascarid species, as orthologues for both *Pun*Pgp-11 paralogues can be found not only in *P. univalens* and *A. suum* but also the more distantly related *T. canis*. Both *Pun*Pgp-16 paralogues have at least orthologues in *A. suum*. Currently, genomic resources for *P. univalens* are extremely limited (only one genome representing a small number of individuals) but it is likely that Pgp evolution is continuously driven through treatment practices. Furthermore, our results indicate that there is a likelihood of alternative splicing in Pgps of nematodes, e.g. as demonstrated for *Pun*Pgp-18. The calculated support (FPKM) for individual exons suggests several alternative splicing events based on present or absent exon support in the different tissues. However, alternate endings of Pgps found in the different contigs and in the annotation as well as alternating support of individual exons in different tissues (Supplementary Fig. S1) could not be confirmed by RT-PCR. Missing exons in the Wang et al., 2017 annotation concentrated at 5′- and 3′ ends (Supplementary Fig. S1) and led to truncated gene models. While we cannot exclude isoforms for Pgps that were not identified with our approach, we were able to significantly improve the annotation of all *P. univalens*. Future improved genome assembly may allow further refinement of the annotation of these genes.

In GenBank, the inventory of nematode Pgps is immense and chaotic, i.e. many published and annotated Pgps including those published for *A. suum* do not adhere to wormbase.org nomenclature guidelines and some genes annotated as hypothetical Pgp are likely to be artefacts of automated assembly and annotation pipelines. Consequently, many of the deposited sequences and gene-models are unreliable and incorrectly named, hindering comparative analysis. Automated annotation and transcriptome resources have immense value. However, as has been demonstrated in model organisms, genome data from next-generation sequencing, automatic assembly and annotation should be validated by independent experimental methods for studies on particular genes and transcripts. Furthermore, transgenic expression and functional analysis as conducted in this study demonstrate that identified protein sequences lead to functional genes.

Considering the efflux mechanism of Pgps, it can be assumed that in order to contribute to resistance a high expression level is necessary. However, from the data provided by the IVM transcriptome dataset, no significant upregulation of any Pgp was detected following IVM treatment. Nonetheless, in a previous study, worms from a resistant *P. univalens* population showed a small but significant higher expression of *Pun*Pgp-11.1 but not of *Pun*Pgp-16.1^44^ but other Pgps were not examined due to the lack of validated Pgp sequences in gene databases. In the present study, the resistance status of IVM incubated worms was unknown and it may be that the worms had a susceptible phenotype, which could be a potential reason for the observed absence of IVM treatment associated inducibility of Pgp genes. From a methodical point of view, it is possible that the IVM concentration and incubation duration are responsible for the encountered lack of significant transcriptomic response or that the artificial culture conditions were unsuited to induce an upregulation of xenobiotic transporters upon IVM encounter in vitro. The experimental set-up has several limitations, for example it does not include a positive control, which is difficult to design since nothing is known about gene regulation in *P. univalens*. Furthermore, some other limitations set by culture conditions to transcriptomes of ascarids derived from in vitro culture were recently revealed^61^. However, with regard to studies of other nematodes e.g. *C. elegans*^62^ or *T. circumcincta*^29^ it appears most likely that constitutively high expression levels of Pgps that can contribute to resistance may be selected over many generations as a heritable trait.

Interestingly, the Pgp intestinal expression by far exceeded that of the other tissues, substantiating evidence for a Pgp-mediated barrier function against uptake of xenobiotics including anthelmintics at the intestine, but this mechanism is not yet understood. As the population of worms used for the tissue specific transcriptome study has never been treated with MLs, it appears that the strong intestinal expression level is innate to *P. univalens.* Likewise, the similarly strong intestinal expression of *C. elegans* Pgps allows the assumption that strong intestinal Pgp expression might be conserved in nematodes, but not at the individual gene level. In *P. univalens*, *Pun*Pgp-9, -11.1 and -16.2 consistently exhibited the strongest expression throughout both independent transcriptome data sets, but in *C. elegans* a different repertoire of high expression Pgp was identified, including Pgp-1 and Pgp-14 which might also be attributed to the different developmental stages. Pgp expression in the carcass tissue (hypodermis, muscle, neuron and pharynx) was second strongest, with the aforementioned set of identical dominant Pgps. It remains unclear whether their protective role to MLs is of any importance in the hypodermis below the cuticle or even directly at the target sites in neurons. To this end, *Pun*Pgp-11.1 and *Pun*Pgp-16.1 mRNA expression was recently detected at the H-shaped excretory system, which forms a continuous canal from the nerve ring to the middle of the body, and the nerve cords. The expression pattern of mRNA expression levels from the same study are in line with our findings^63^. Strikingly, in a previous study, a *C. elegans* strain with a loss-of-function allele of the predominantly pharyngeally expressed *Cel*Pgp-14 showed the strongest IVM susceptibility increase of all *C. elegans* Pgp mutant lines in a development assay^51^. Moreover, this Pgp was upregulated in an IVM resistant (avr-14/avr-15/glc-1 deficient) *C. elegans* strain^64^, suggesting that Pgp expression at the pharynx could protect relevant regulatory neurons and alter IVM susceptibility. Further investigations on the mechanism and role of tissue specific ABC-transporter expression in nematodes are needed, e.g. tissue specific transcriptomic analysis upon ML or other anthelmintic exposure as small to moderate gene expression fold changes of individual genes can be missed when analysing whole worm samples.

To compare the Pgp transport potential of important anthelmintics, two previously resistance associated Pgp orthologues were chosen for functional characterization, with *Pun*Pgp-2 being a moderate expression Pgp and *Pun*Pgp-9 a high expression Pgp. Transgenic expression of *Pun*Pgp-11.1 was previously shown to decrease IVM susceptibility of *C. elegans*^52^. Here, we show that both *Pun*Pgp-2 and *Pun*Pgp-9 can also interact with IVM. This confirms previous reports^47–49,51,53,55^ that different Pgp orthologues can transport MLs (in this case IVM) which also explains why following selection pressure different Pgp and other ABC-transporters underwent expression changes depending on the study and the nematode species^56,62,65^. Our results support that both *Pun*Pgp-2 and *Pun*Pgp-9 are transmembrane transporters and excrete KCON and IVM out of the yeast cells. Notably, for Pgp to effectively reduce the effective concentration of a toxin or drug in nematodes, for example by efflux in the intestine, their localisation and predominant efflux activity at the apical intestinal membrane is a prerequisite. Conversely, localisation at the baso-lateral surface would result in influx and thus increased susceptibility. For example, *C. elegans* Pgp-1 was shown to localize exclusively to the apical membrane^66^. In addition to their role in ML detoxification, Pgps have been linked to resistance against other anthelmintics such as benzimidazoles^67^ and more recently monepantel^68^. However, the results of the present study do not indicate an interaction between either *Pun*Pgp-2 or *Pun*Pgp-9 and the benzimidazole derivative TBZ. Nonetheless, the demonstrated interaction between both Pgps and the structurally unrelated KCON and IVM show that these Pgps are indeed multi-drug transporters with a wide substrate range and their interaction with monepantel remains to be elucidated. With respect to the conserved large repertoire of Pgps in nematodes, it also appears likely that several Pgps are also involved in endogenous physiological processes. For instance, *Cel*Pgp-2 has been linked to lipid storage^69^ and in *P. univalens*, *Pun*Pgp-10 and -11.2 expression is focused to the ovary (Fig. 3a), suggesting a role in reproductive processes. Noteworthy, while Pgp-2 orthologues have an important physiological function in *C. elegans*, the results of this study show the broad substrate range of a Pgp-2 orthologue which results in functional flexibility. Therefore, it can be speculated that evolutionary changes in the life cycle and behaviour accompanied by changes in tissue expression patterns alter the actual function and role of a Pgp orthologue. In the context of resistance and with respect to the previously mentioned differences in the Pgp repertoire implicated with ML resistance in different nematode species, it is likely that the Pgp expression pattern in target and barrier tissues of individual Pgp in a specific nematode population at the time of treatment is a major confounding factor influencing which Pgp contribute to ML resistance.

In conclusion, this study provides a valuable resource for Pgp-mediated anthelmintic resistance research through improved and reliable gene models from experimentally verified Pgp sequences in *P. univalens.* Furthermore, the analysis of tissues specific expression level and characterization of the interaction of two *P. univalens* Pgps with two important anthelmintic classes represented by IVM and TBZ, improve the foundation for understanding Pgp-mediated anthelmintic resistance. To this end, there is an urgent need to improve the understanding of how individual Pgp orthologues interact with different ML derivatives and contribute to ML resistance as well as to elucidate the functional role of tissue specific expression of individual Pgps.

## Materials and methods

### Biological material, genome and transcriptome resources

Two independent transcriptome data sets of *P. univalens* were used in this study.

A previously published total RNA transcriptome data set (GeoDataset accession: GSE99524; GenBank transcriptome assembly accession: GCA_002259205.1 WormBase ParaSite version WPBS14) was used which included samples with two replicates each of male and female gonads, embryo, intestine, carcass (remainder after dissection of intestine and germline tissue including pharynx, hypodermis, muscle and neuronal tissue) as well as a male and female mixed sample, the latter consisting of a 5′-untranslated region (UTR) spliced leader PCR library and a 5′-UTR TeloPrime PCR library^56^, referred to as tissue specific transcriptome. Originating from the same study, a reference genome assembly (GenBank assembly accession: genome assembly ASM225920v1, version WBPS14) and an *Ascaris suum* transcriptome assembly (GenBank transcriptome assembly: GCA_000187025.3) were used^56^. The corresponding annotation of the *P. univalens* reference genome assembly was obtained from WormBase ParaSite, version WBPS14. The worms for this study are from a research herd maintained at the University of Kentucky with no anthelmintic usage since 1979, which means no prior exposure to MLs^70^.

A second total RNA transcriptome data set was generated from a total of 10 individual *P. univalens* adult worms, referred to as IVM-transcriptome. All *P. univalens* were collected from an abattoir in Kraków and their resistance status and treatment history was unknown. For in vitro culture, only female worms were selected and handled as described previously^44^. In brief, worms were incubated at 37 °C in artificial perienteric fluid (APF, 5 mM MgCl_2_, 6 mM CaCl_2_, 24 mM KCl, 23 mM NaCl, 110 mM NaCH_3_COO, 11 mM dextrose, 10 mM Tris and was adjusted to a final pH of 7.5 at 37 °C) in ventilated tissue culture flasks, 1–2 worms per flask, for 18 h. Then, 10 worms were further incubated in pairs of two in fresh APF containing 10^–9^ M IVM (Sigma-Aldrich) for twelve hours while 10 control worms were incubated only in the presence of the vehicle (1% DMSO). After incubation, individual worms were rapidly frozen on dry ice and stored at − 80 °C until use. RNA was isolated from all worms using the whole worm and TriFast reagent (Peqlab). Before further use, quality of all RNAs was controlled on the Bioanalyzer 2,100 (Agilent) with the RNA 6,000 Nano Kit. All RNAs used in the experiments had RNA integrity numbers (RIN) ≥ 9.1. In order to confirm the sex of worms as obtained using morphological criteria, an RT-PCR targeting the vitellogenin-6 RNA was conducted and only vit-6 positive samples were included^44^. For deep-sequencing, five control and five samples from IVM incubated worms were chosen. After construction of 10 individual (barcoded), random-primed libraries for paired-end sequencing, all libraries were sequenced together on a single lane of an Illumina HiSeq 2000 to obtain 100 bp paired end reads. CASAVA software version 1.8.2 (Illumina) was used to demultiplex all samples and to clip adapters from all reads. Ribosomal RNA sequences were identified and filtered out using RiboPicker 0.4.3^71^. Data were quality filtered by (i) removing all reads containing more than one N, (ii) removal of bases or complete reads containing sequencing errors, (iii) trimming of reads at the 3′-end to obtain a minimum average Phred quality ≥ 10 over a window of 10 bases and (iv) discarding all reads with less than 20 bases left after the above quality adjustments. Worms from the same population as the IVM-transcriptome were^44^ used for RNA extraction for RT-PCR.

*Saccharomyces cerevisiae* strain AD1234567 (AD1-7) deficient in seven endogenous ABC-transporters^72^ was kindly provided by André Goffeau (Université Catholique de Louvain, Belgium). The strain was maintained and grown at 30 °C in Yeast Extract-Peptone-Dextrose (YPD) medium plates (10 g yeast extract, 20 g peptone, 20 g dextrose, 15–20 g Agar in 1 L H_2_O bidest, (Sigma-Aldrich)) at 250 rpm or on YPD agar plates with 15–25 g/L additional agar.

All reagents were purchased from Thermo Fisher Scientific unless stated otherwise.

### Amplification and cloning of full length P-glycoproteins

To identify contigs encoding ABCB transporters, both transcriptomes were analysed with NCBI software Basic Local Alignment Search Tool (BLAST)^73^ TBLASTN using *P. univalens* Pgp-11.1 and Pgp-16.1 protein sequences (GenBank accession numbers in Supplementary Table S3) as query. To examine full gene coverage, the identified contigs were then compared to the other nematode Pgps in the GenBank database using BLASTX. For each putative Pgp, the contig or a combination of contigs with the highest coverage of a complete Pgp was used for the following steps, while putative half-transporters were excluded. Gene specific primers for RT-PCR were designed manually from 3′- and 5′- UTR of all potential alternative endings for amplification of full length Pgps. For contigs spanning merely a fraction of a Pgp gene, primer pairs were designed at the 5′- and 3′-ends of each sequence (all primers in Supplementary Table S2).

First strand cDNA was synthesized from 1–2 μg of total RNA using the Maxima H Minus First Strand cDNA Synthesis Kit according to the manufacturer’s instructions. The PCR reaction mixture contained ca. 1–2 ng/µL cDNA, 0.02 U/µl Phusion Hot Start II polymerase, 1 M betaine (Sigma-Aldrich), each primer at 0.5 μM and each dNTP at 200 μM in 25 µL 1 × HF buffer. An initial denaturation at 98 °C for 30 s was followed by 35 cycles of denaturation at 98 °C for 10 s, annealing at a primer pair specific Tm (Supplementary Table S2) for 30 s and elongation at 72 °C for fragment specific elongation time (Supplementary Table S2). A final elongation was performed at 72 °C for 300 s. Purified PCR products were directly cloned using the StrataClone Blunt PCR Cloning Kit (Agilent) according to the manufacturer’s instructions. Extracted plasmids (ZymoPURE II Plasmid Midiprep Zymo Research) were verified by restriction analysis and sequenced by primer walking technique (LGC Genomics).

In case of missing transcriptome sequence data at the 5′- or 3′-end, contig sequences were first verified by RT-PCR as described earlier followed by the 5′-end amplification by nested RT-PCR using a primer targeting the *A. suum* spliced leader sequence^74^ as a forward primer and two gene specific reverse primers. 3′-end amplification was performed using the 5′/3′ RACE Kit, 2nd Generation (Roche) according to the manufacturer’s instructions (all primers Supplementary Table S2).

### Phylogenetic analysis

Pgp protein sequences of nematodes and a representative outgroup were obtained from GenBank and WormBase (GenBank accession numbers or WormBase IDs in Supplementary Table S3). Additionally, *A. suum* transcripts were obtained through BLASTX analysis of the latest transcriptome assembly using *P. univalens* cDNA sequences as query. Open reading frames were then manually corrected using the *P. univalens* orthologue as a template. Alignment was carried out using M-Coffee^75–78^ with default parameters. For determination of an optimal amino acid substitution model, the alignment was analysed with ProtTest 3.0^79^. Thereafter, a consensus tree was calculated using RAxML 8.2.9^80^ and the LG + F + G model^81^. with 1,000 bootstrap replicates allowing both nearest neighbour interchange (NNI) and subtree pruning and regraftment (SPR) moves. Using the tree obtained by bootstrapping to restrict the tree topology, the Shimodaira-Hasegawa (SH) approximate likelihood ratio test was used to calculate additional branch support values in RAxML. To prevent potential analysis errors due to the high variability in certain sequence regions, a second tree was computed following analysis and editing of the original alignment with GBLOCKS 0.91b^82^ with default parameters to exclude highly variable positions. Finally, the best tree was visualized using MEGA 7^83^ rooting the tree using the outgroup.

### Re-annotation of the *Parascaris univalens* genome and expression analyses

Genomic coordinates of intron and exon borders were predicted in the *P. univalens* genome (assembly ASM225920v1, WormBase ParaSite version WPBS14) based on mapping of the cDNA sequences using the NCBI tool Splign (version 2.1.0; based on compart version 1.35)^84^. Coordinates were converted to gff3 format and used to “patch” the genome annotation (removing the original annotation in the overlapping areas). Mapping of raw reads of individual sample data sets against the genome was conducted using STAR (version 2.5.4b)^85^ and expression levels of Pgps were estimated using featureCounts^86^ against the patched annotation. Resulting raw counts were normalised for the overall size of the sequencing library as Transcripts per Kilobase Million (TPM).

TPM expression levels were visualized with GraphPad Prism version 8.3.3 (GraphPad Software, San Diego, California USA, https://www.graphpad.com). For the IVM incubated transcriptome, each data point was visualized and differences in expression levels of individual Pgp were analysed with the Kruskal–Wallis test followed by a Dunn’s Post-Hoc (α = 0.05) test with corrections for multiple testing. For the tissue specific transcriptome data set, the mean of two replicates was visualized and no statistical analysis was conducted. Finally, single cell transcriptome was used to compare tissue expression levels, calculated with R^87^ package monocle3^88–90^ of individuals Pgps in *C. elegans* L2^58^ (Geo Accession number Accession: GSE98561). For visualization of low expression tissues, expression was visualized on a log_10_ scale and for better comparison of overall expression levels, on a linear scale. Mappings (bam files from STAR) were also analysed using the R package SGSeq^91^ to visualize coverage of individual exons (as FPKM) in a heatmap. For comparison of the new annotation to the original Pgp annotations from Wang et al., 2018 (WormBase ParaSite version WPBS14), both annotations were visualized with Geneious Prime 2019.2.3 (https://www.geneious.com) and then combined with exon coverage heatmaps of the experimental cDNA exons using CoralDRAW X7 (https://www.coraldraw.com).

### Plasmid assembly

cDNA sequences of *Pun*Pgp-2 and *Pun*Pgp-9 were codon optimized for expression in *S. cerevisiae* and synthesized excluding the stop codon (BioCat GmbH) (sequences in Supplementary Table S2) in two overlapping parts and then assembled by PCR. For each gene, a reaction mixture was prepared containing the two fragments at equimolar concentrations of 5 fmol each along with 0.02 U/µL Phusion Hot Start II polymerase (Thermo Fisher Scientific), each dNTP at 200 μM in 25 µL 1 × HF buffer. Denaturation at 98 °C for 30 s was followed by 12 cycles each of denaturation at 98 °C for 10 s, annealing at 68 °C for 30 s and elongation at 72 °C for 200 s. Then forward and reverse primers (Supplementary Table S2) were added at 0.5 µM to 28 µL total volume in 1 × HF buffer matching the 5′ and 3′ end of the sequence and cycled 35 times under the same conditions and ending with a 5 min elongation. Purified PCR products were incubated with Taq DNA Polymerase (Thermo Fisher Scientific) in 20 µL of 1 × Taq DNA Polymerase Buffer, 2 µL MgCl_2_ for 72 °C at 20 min to add 3′ desoxyadenosine overhangs. Then, products were purified and ligated into the pYes2.1 vector followed by transformation using the pYES2.1 TOPO TA Yeast Expression Kit (Thermo Fisher Scientific) according to the manufacturer’s instructions. Sequences of plasmids extracted from *Escherichia coli* Top10 cultures were validated by Sanger sequencing (LGC Genomics).

### Transformation into *Saccharomyces cerevisiae*

pYES2.1-his plasmids containing *Pun*Pgp-2 and *Pun*Pgp-9 or the β-galactosidase gene (lacZ) (supplied control vector) were transfected by the lithium acetate method into AD1-7 yeast using the pYES2.1 TOPO TA Yeast Expression Kit (Thermo Fisher Scientific) according to the manufacturer’s instructions. Successfully transformed yeast were grown for 48 h on synthetic minimal medium plates lacking uracil (SC-U) (6.7 g/L yeast nitrogen base with amino acids, 1.92 g/L yeast drop-out medium supplement without uracil, 2% glucose, 1% raffinose and 2% agar, omitting agar when making synthetic minimal medium, see recipes in user manual pYES2.1 TOPO TA Yeast Expression Kit, Sigma-Aldrich).

### Verification of expression by RT-PCR

Transcription of *Pun*Pgp-2 and *Pun*Pgp-9 was analysed by RT-PCR. Yeast were grown in induction media containing galactoses (SC-U medium but substituting the carbon source to 2% galactose and 1% raffinose) which induces activation of the GAL1 promotor and results in expression of the inserted gene. After 24 h of incubation at 30 °C and 250 rpm, total RNA was extracted using the Maxwell Simply RNA kit (Promega). cDNA was synthesized using the Maxima H- First Strand cDNA Synthesis Kit (Thermo Fisher Scientific) from ~ 1 µg of RNA using Oligo(dT)_18_Primers according to the manufacturer’s instructions. RT-PCR was performed using 0.02 U/µL Phusion Hot Start II polymerase (Thermo Fisher Scientific), 0.5 µM each primer, each dNTP at 200 μM, 1–2 ng/µL cDNA in a total volume of 25 µL 1 × HF buffer for two different primer sets (Primers in Supplementary Table S2). After a denaturation at 98 °C, 35 cycles of 98 °C for 10 s, a primer pair-specific annealing temperature for 15 s and 72 °C for 30 s were performed. Amplified PCR product were visualised after gel electrophoresis with GrGreen.

### Yeast growth inhibition assay

Yeast growth inhibition assays were performed in principle as explained elsewhere^47,92^. Briefly, prior to the actual assay transgene expression of *Pun*Pgp-2, *Pun*Pgp-9 or the lacZ gene was induced in AD1-7 *S. cerevisiae* strain by growing yeast in 5 mL induction media at 30 °C and 250 rpm for 24 h. Then, after harvesting the yeast by centrifugation, yeast cells were seeded at 4 × 10^4^ cells/well into a 96 well plate in a final volume of 100 µL induction medium containing drugs as described below dissolved in DMSO at a final concentration of 1% (IVM and KCON) or directly in water (TBZ). The plates were sealed with parafilm to avoid concentrations shifts due to evaporation and incubated in the Synergy 4 plate reader (Biotech) for 48 h at 30 °C and 250 rpm shaking. Every 10 min, shaking was interrupted and absorption at 600 nm (OD_600_) was measured.

To determine the susceptibility of yeast strains to KCON and TBZ, yeast were incubated with a dilution series of KCON (2.94 µM, 2.2 µM, 1.46 µM, 1.09 µM, 0.37 µM and 0.18 µM, 0.00 µM KCON) at a final total 1% DMSO concentration or TBZ (2.4 mM, 1.2 mM, 0.6 mM, 0.3 mM, 0.15 mM, 0 mM TBZ) without DMSO generating a minimum of 12 replicates split equally on three separate days. For *Pun*Pgp-2 and *Pun*Pgp-9 expressing strains the maximum tolerated KCON concentration of 0.4 µM was chosen and co-incubated with an IVM dilution series (0.0 µM, 0.1 µM, 0.2 µM, 0.4 µM, 0.73 µM, 1.5 µM, 2.9 µM, 4.4 µM, 5.9 µM IVM) at a final total 1% DMSO concentration and also generating a minimum of 12 replicates split equally on three separate days.

Analysis was done similarly as described elsewhere^47^ and all calculations and visualizations were conducted using GraphPad Prism 8.3.0 unless specified otherwise. In brief, raw absorption data which correlates with yeast growth was exported from Gen5 Data Analysis Software (BioTek), blanked to wells containing no yeast and only medium and excluding the first 50 measurements. Then, the area under the curve (AUC) was calculated for each well. To determine relative growth, the AUC was then normalised to the mean AUC of the negative controls (containing no TBZ, no KCON but 1% DMSO or IVM or for IVM + KCON co-incubation a fixed KCON concentration) of the same yeast strain and on the same day. For non-linear regression analysis concentrations were log_10_ transformed. For this, the 0 M concentration (no drug negative controls) were set to 0.1 µM KCON and 0.7 µM TBZ. To determine susceptibility to directly antimycotic substances KCON and TBZ, four parameter non-linear regression models (model: Y = Bottom + (Top − Bottom)/(1 + 10^((Log EC_50_ − X)*Hillslope)) were calculated from a minimum of 12 replicates per concentration and due to the normalisation to relative growth top values were constrained to a maximum of 1 and bottom values to a minimum of 0. From the non-linear regression models, half maximal effective concentrations (EC_50_) and corresponding 95% confidence intervals as well as R^2^ values as a determinant for goodness of fit were calculated. To compare susceptibility of AD1-7Pgp-2 and AD1-7Pgp-9 to AD1-7lacZ, the extra sum-of-squares F test for the LogEC_50_ was used and p-values were corrected for multiple testing with the Holm’s method in R version 3.6.2^87^, considering corrected p-values > 0.05 as significant.

The direct inhibitory effect of IVM in the presence or absence of yeast was analysed in R. First, direct effect of IVM on yeast growth was analysed for using a one-way analysis of variance (ANOVA) followed by a Tukey’s HSD post-hoc test with Holm’s p-value adjustment. Then, using a two-way ANOVA the effect on yeast growth of IVM concentration (factor 1) in the absence or presence of a fixed KCON concentration (factor 2) was analysed. To analyse differences between the absence and presence of KCON at each IVM concentration within each strain, a Dunn’s post-hoc test^93^ was conducted with Holm’s p-value adjustment. For all analyses, an adjusted p-value less than 0.05 was considered significant.

## Supplementary information

Supplementary information

## Data Availability

BankIt2307849 PunPgp-2: MT001899, BankIt2307849 PunPgp-3: MT001900, BankIt2307849 PunPgp-9: MT001901, BankIt2307849 PunPgp-10: MT001902, BankIt2307849 PunPgp-11.2: MT001904, BankIt2307849 PunPgp-12: MT001905, BankIt2307849 PunPgp-16.2: MT001907, BankIt2307849 PunPgp-18A: MT001908, BankIt2307849 PunPgp-18B: MT001909. The updated annotation (gff3) of P-glycoproteins has been shared with WormBase ParaSite and will be available on the next WormBase Parasite release.

## References

[1] Cribb NC, Cote NM, Boure LP, Peregrine AS (2006). Acute small intestinal obstruction associated with Parascaris equorum infection in young horses: 25 cases (1985–2004). N. Z. Vet. J..

[2] Nielsen MK (2016). Evidence-based considerations for control of *Parascaris* spp. infections in horses. Equine Vet. Educ..

[3] Kaplan RM (2004). Drug resistance in nematodes of veterinary importance: a status report. Trends Parasitol..

[4] von Samson-Himmelstjerna G (2012). Anthelmintic resistance in equine parasites - detection, potential clinical relevance and implications for control. Vet. Parasitol..

[5] Prichard RK, Hall CA, Kelly JD, Martin ICA, Donald AD (1980). The problem of anthelmintic resistance in nematodes. Aust. Vet. J..

[6] von Samson-Himmelstjerna G (2009). Effects of worm control practices examined by a combined faecal egg count and questionnaire survey on horse farms in Germany, Italy and the UK. Parasites Vectors.

[7] Boersema JH, Eysker M, Nas JW (2002). Apparent resistance of *Parascaris equorum* to macrocylic lactones. Vet. Rec..

[8] N. Kettner, H. H. In *Annual meeting of the Deutsche Veterinärmedizinische Gesellschaft Fachgruppe "Parasitologie und parasitäre Krankheiten"* (Hannover, 2017).

[9] Näreaho A, Vainio K, Oksanen A (2011). Impaired efficacy of ivermectin against *Parascaris equorum*, and both ivermectin and pyrantel against strongyle infections in trotter foals in Finland. Vet. Parasitol..

[10] Relf VE, Lester HE, Morgan ER, Hodgkinson JE, Matthews JB (2014). Anthelmintic efficacy on UK Thoroughbred stud farms. Int. J. Parasitol..

[11] von Samson-Himmelstjerna G (2007). Cases of reduced cyathostomin egg-reappearance period and failure of *Parascaris equorum* egg count reduction following ivermectin treatment as well as survey on pyrantel efficacy on German horse farms. Vet. Parasitol..

[12] Lassen B, Peltola SM (2015). Anthelmintic resistance of intestinal nematodes to ivermectin and pyrantel in Estonian horses. J. Helminthol..

[13] Schougaard H, Nielsen MK (2007). Apparent ivermectin resistance of *Parascaris equorum* in foals in Denmark. Vet. Rec..

[14] Slocombe JO, de Gannes RV, Lake MC (2007). Macrocyclic lactone-resistant *Parascaris equorum* on stud farms in Canada and effectiveness of fenbendazole and pyrantel pamoate. Vet. Parasitol..

[15] Lyons ET, Tolliver SC, Ionita M, Collins SS (2008). Evaluation of parasiticidal activity of fenbendazole, ivermectin, oxibendazole, and pyrantel pamoate in horse foals with emphasis on ascarids (*Parascaris equorum*) in field studies on five farms in Central Kentucky in 2007. Parasitol. Res..

[16] Craig TM, Diamond PL, Ferwerda NS, Thompson JA (2007). Evidence of Ivermectin Resistance by *Parascaris equorum* on a Texas Horse Farm. J. Equine Vet. Sci..

[17] Bishop RM (2014). Sub-optimal efficacy of ivermectin against *Parascaris equorum* in foals on three Thoroughbred stud farms in the Manawatu region of New Zealand. N. Z. Vet. J..

[18] Armstrong SK (2014). The efficacy of ivermectin, pyrantel and fenbendazole against *Parascaris equorum* infection in foals on farms in Australia. Vet. Parasitol..

[19] Seyoum Z, Zewdu A, Dagnachew S, Bogale B (2017). Anthelmintic resistance of strongyle nematodes to ivermectin and fenbendazole on cart horses in Gondar, Northwest Ethoipia. Biomed. Res. Int..

[20] Alanazi AD (2017). A field study on the anthelmintic resistance of *Parascaris* spp. in Arab foals in the Riyadh region, Saudi Arabia. Vet. Q..

[21] Sutherland IA, Brown AE, Leathwick DM, Bisset SA (2003). Resistance to prophylactic treatment with macrocyclic lactone anthelmintics in *Teladorsagia circumcincta*. Vet. Parasitol..

[22] Kotze AC, Prichard RK (2016). Anthelmintic resistance in *Haemonchus contortus*: history, mechanisms and diagnosis. Adv. Parasitol..

[23] Bourguinat C (2015). Macrocyclic lactone resistance in *Dirofilaria immitis*: Failure of heartworm preventives and investigation of genetic markers for resistance. Vet. Parasitol..

[24] Osei-Atweneboana MY, Eng JK, Boakye DA, Gyapong JO, Prichard RK (2007). Prevalence and intensity of Onchocerca volvulus infection and efficacy of ivermectin in endemic communities in Ghana: a two-phase epidemiological study. Lancet.

[25] Whittaker JH, Carlson SA, Jones DE, Brewer MT (2017). Molecular mechanisms for anthelmintic resistance in strongyle nematode parasites of veterinary importance. J. Vet. Pharmacol. Ther..

[26] Gilleard JS (2006). Understanding anthelmintic resistance: the need for genomics and genetics. Int. J. Parasitol..

[27] James CE, Hudson AL, Davey MW (2009). Drug resistance mechanisms in helminths: is it survival of the fittest?. Trends Parasitol..

[28] Doyle SR (2017). Genome-wide analysis of ivermectin response by *Onchocerca volvulus* reveals that genetic drift and soft selective sweeps contribute to loss of drug sensitivity. PLoS Negl. Trop. Dis..

[29] Choi YJ (2017). Genomic introgression mapping of field-derived multiple-anthelmintic resistance in *Teladorsagia circumcincta*. PLoS Genet..

[30] Khan S (2020). A whole genome re-sequencing based GWA analysis reveals candidate genes associated with ivermectin resistance in *Haemonchus contortus*. Genes..

[31] Rezansoff AM, Laing R, Gilleard JS (2016). Evidence from two independent backcross experiments supports genetic linkage of microsatellite Hcms8a20, but not other candidate loci, to a major ivermectin resistance locus in *Haemonchus contortus*. Int. J. Parasitol..

[32] Doyle SR (2018). A major locus for ivermectin resistance in a parasitic nematode. bioRxiv.

[33] Jones PM, George AM (2004). The ABC transporter structure and mechanism: perspectives on recent research. Cell. Mol. Life Sci..

[34] Hodges LM (2011). Very important pharmacogene summary: ABCB1 (MDR1, P-glycoprotein). Pharmacogenet. Genom..

[35] Hooiveld GJEJ (2002). Stereoselective transport of hydrophilic quaternary drugs by human *MDR1* and rat *Mdr1b* P-glycoproteins. Br. J. Pharmacol..

[36] Aller SG (2009). Structure of P-glycoprotein reveals a molecular basis for poly-specific drug binding. Science.

[37] Annilo T (2006). Evolution of the vertebrate ABC gene family: analysis of gene birth and death. Genomics.

[38] Sheps JA, Ralph S, Zhao Z, Baillie DL, Ling V (2004). The ABC transporter gene family of *Caenorhabditis elegans* has implications for the evolutionary dynamics of multidrug resistance in eukaryotes. Genome Biol..

[39] Zhao Z, Sheps JA, Ling V, Fang LL, Baillie DL (2004). Expression analysis of ABC transporters reveals differential functions of tandemly duplicated genes in *Caenorhabditis elegans*. J. Mol. Biol..

[40] Lespine A, Alvinerie M, Vercruysse J, Prichard RK, Geldhof P (2008). ABC transporter modulation: a strategy to enhance the activity of macrocyclic lactone anthelmintics. Trends Parasitol..

[41] Croop JM (1993). P-glycoprotein structure and evolutionary homologies. Cytotechnology.

[42] Roulet, A. & Prichard, R. K. In *Annual Meeting of the American Association of Veterinary Parasitologists, Abstract No 72* (Honolulu, USA, 2006)

[43] Dicker AJ, Nisbet AJ, Skuce PJ (2011). Gene expression changes in a P-glycoprotein (*Tci*-pgp-9) putatively associated with ivermectin resistance in *Teladorsagia circumcincta*. Int. J. Parasitol..

[44] Janssen IJ (2013). Genetic variants and increased expression of *Parascaris equorum* P-glycoprotein-11 in populations with decreased ivermectin susceptibility. PLoS ONE.

[45] Turnbull F, Jonsson NN, Kenyon F, Skuce PJ, Bisset SA (2018). P-glycoprotein-9 and macrocyclic lactone resistance status in selected strains of the ovine gastrointestinal nematode, *Teladorsagia circumcincta*. Int. J. Parasitol. Drugs Drug Resist..

[46] Xu M (1998). Ivermectin resistance in nematodes may be caused by alteration of P-glycoprotein homolog. Mol. Biochem. Parasitol..

[47] Kaschny M (2015). Macrocyclic lactones differ in interaction with recombinant P-glycoprotein 9 of the parasitic nematode *Cylicocylus elongatus* and ketoconazole in a yeast growth assay. PLoS Pathog..

[48] Godoy P, Che H, Beech RN, Prichard RK (2015). Characterization of *Haemonchus contortus* P-glycoprotein-16 and its interaction with the macrocyclic lactone anthelmintics. Mol. Biochem. Parasitol..

[49] Godoy P, Lian J, Beech RN, Prichard RK (2015). *Haemonchus contortus* P-glycoprotein-2: in situ localisation and characterisation of macrocyclic lactone transport. Int. J. Parasitol..

[50] David M (2016). In silico analysis of the binding of anthelmintics to *Caenorhabditis elegans* p-glycoprotein 1. Int. J. Parasitol. Drugs Drug Resist..

[51] Janssen IJ, Krucken J, Demeler J, von Samson-Himmelstjerna G (2013). *Caenorhabditis elegans*: modest increase of susceptibility to ivermectin in individual P-glycoprotein loss-of-function strains. Exp. Parasitol..

[52] Janssen IJ, Krucken J, Demeler J, von Samson-Himmelstjerna G (2015). Transgenically expressed *Parascaris* P-glycoprotein-11 can modulate ivermectin susceptibility in *Caenorhabditis elegans*. Int. J. Parasitol. Drugs Drug Resist..

[53] Mani T (2016). Interaction of macrocyclic lactones with a *Dirofilaria immitis* P-glycoprotein. Int. J. Parasitol..

[54] Godoy P, Lian J, Beech RN, Prichard RK (2015). *Haemonchus contortus* P-glycoprotein-2: in situ localisation and characterisation of macrocyclic lactone transport. Int. J. Parasitol..

[55] Godoy P, Che H, Beech RN, Prichard RK (2016). Characterisation of P-glycoprotein-9.1 in *Haemonchus contortus*. Parasites Vectors.

[56] Wang J (2017). Comparative genome analysis of programmed DNA elimination in nematodes. Genome Res..

[57] Blaxter ML (1998). A molecular evolutionary framework for the phylum Nematoda. Nature.

[58] Cao J (2017). Comprehensive single-cell transcriptional profiling of a multicellular organism. Science.

[59] Laing R (2013). The genome and transcriptome of *Haemonchus contortus*, a key model parasite for drug and vaccine discovery. Genome Biol..

[60] Ardelli BF, Stitt LE, Tompkins JB (2010). Inventory and analysis of ATP-binding cassette (ABC) systems in *Brugia malayi*. Parasitology.

[61] Scare JA (2020). Ascarids exposed: a method for in vitro drug exposure and gene expression analysis of anthelmintic naïve *Parascaris* spp. Parasitology.

[62] Figueiredo LA (2018). Dominance of P-glycoprotein 12 in phenotypic resistance conversion against ivermectin in Caenorhabditis elegans. PLoS ONE.

[63] Jesudoss Chelladurai J, Brewer MT (2019). Detection and quantification of *Parascaris* P-glycoprotein drug transporter expression with a novel mRNA hybridization technique. Vet. Parasitol..

[64] Ardelli BF, Prichard RK (2013). Inhibition of P-glycoprotein enhances sensitivity of *Caenorhabditis elegans* to ivermectin. Vet. Parasitol..

[65] De Graef J (2013). Gene expression analysis of ABC transporters in a resistant *Cooperia oncophora* isolate following in vivo and in vitro exposure to macrocyclic lactones. Parasitology.

[66] Sato T (2007). The Rab8 GTPase regulates apical protein localization in intestinal cells. Nature.

[67] Blackhall WJ, Prichard RK, Beech RN (2008). P-glycoprotein selection in strains of *Haemonchus contortus* resistant to benzimidazoles. Vet. Parasitol..

[68] Raza A, Bagnall NH, Jabbar A, Kopp SR, Kotze AC (2016). Increased expression of ATP binding cassette transporter genes following exposure of *Haemonchus contortus* larvae to a high concentration of monepantel in vitro. Parasites Vectors.

[69] Schroeder LK (2007). Function of the *Caenorhabditis elegans* ABC transporter PGP-2 in the biogenesis of a lysosome-related fat storage organelle. Mol. Biol. Cell.

[70] Lyons ET, Drudge JH, Tolliver SC (1990). Prevalence of some internal parasites found (1971–1989) in horses born on a farm in central kentucky. J. Equine Vet. Sci..

[71] Schmieder R, Lim YW, Edwards R (2012). Identification and removal of ribosomal RNA sequences from metatranscriptomes. Bioinformatics.

[72] Portnoy ME, Schmidt PJ, Rogers RS, Culotta VC (2001). Metal transporters that contribute copper to metallochaperones in Saccharomyces cerevisiae. Mol. Genet. Genom..

[73] Altschul SF, Gish W, Miller W, Myers EW, Lipman DJ (1990). Basic local alignment search tool. J. Mol. Biol..

[74] Nilsen TW (1989). Characterization and expression of a spliced leader RNA in the parasitic nematode *Ascaris lumbricoides* var. *suum*. Mol. Cell. Biol..

[75] Moretti S (2007). The M-Coffee web server: a meta-method for computing multiple sequence alignments by combining alternative alignment methods. Nucleic Acids Res..

[76] Notredame C, Higgins DG, Heringa J (2000). T-Coffee: A novel method for fast and accurate multiple sequence alignment. J. Mol. Biol..

[77] Di Tommaso P (2011). T-Coffee: a web server for the multiple sequence alignment of protein and RNA sequences using structural information and homology extension. Nucleic Acids Res..

[78] Wallace IM, O'Sullivan O, Higgins DG, Notredame C (2006). M-Coffee: combining multiple sequence alignment methods with T-Coffee. Nucleic Acids Res..

[79] Darriba D, Taboada GL, Doallo R, Posada D (2011). ProtTest 3: fast selection of best-fit models of protein evolution. Bioinformatics.

[80] Stamatakis A (2014). RAxML version 8: a tool for phylogenetic analysis and post-analysis of large phylogenies. Bioinformatics.

[81] Le SQ, Gascuel O (2008). An improved general amino acid replacement matrix. Mol. Biol. Evol..

[82] Castresana J (2000). Selection of conserved blocks from multiple alignments for their use in phylogenetic analysis. Mol. Biol. Evol..

[83] Kumar S, Stecher G, Tamura K (2016). MEGA7: molecular evolutionary genetics analysis version 7.0 for bigger datasets. Mol. Biol. Evol..

[84] Kapustin Y, Souvorov A, Tatusova T, Lipman D (2008). Splign: algorithms for computing spliced alignments with identification of paralogs. Biol. Direct.

[85] Dobin A (2013). STAR: ultrafast universal RNA-seq aligner. Bioinformatics.

[86] Liao Y, Smyth GK, Shi W (2014). featureCounts: an efficient general purpose program for assigning sequence reads to genomic features. Bioinformatics.

[87] R Core Team. *R: A Language and Environment for Statistical Computing*. Vienna, Austria. https://www.R-project.org/. (2019)

[88] Qiu X (2017). Reversed graph embedding resolves complex single-cell trajectories. Nat. Methods.

[89] Qiu X (2017). Single-cell mRNA quantification and differential analysis with Census. Nat. Methods.

[90] Trapnell C (2014). The dynamics and regulators of cell fate decisions are revealed by pseudotemporal ordering of single cells. Nat. Biotechnol..

[91] Goldstein LD (2016). Prediction and quantification of splice events from RNA-Seq Data. PLoS ONE.

[92] Toussaint M, Conconi A (2006). High-throughput and sensitive assay to measure yeast cell growth: a bench protocol for testing genotoxic agents. Nat. Protoc..

[93] Dinno, A. (2017). dunn.test: Dunn’s Test of multiple comparisons using rank sums. v. R package version 1.3.5, https://CRAN.R-project.org/package=dunn.test.

