# Supplementary material for: The P-glycoprotein repertoire of the equine parasitic nematode Parascaris univalens
Source: Sci Rep. 2020 Aug 12;10:13586. doi: 10.1038/s41598-020-70529-6 (PMC7423980; doi:10.1038/s41598-020-70529-6)
Supplement: Supplementary file 1 — Supplementary information [file 41598_2020_70529_MOESM1_ESM.pdf]

The P-glycoprotein repertoire of the equine parasitic nematode *Parascaris univalens*

Alexander P. Gerhard[1], Jürgen Krücken[1], Emmanuel Heitlinger[2,3], I. Jana I. Janssen[1], Marta Basiaga[4], Sławomir Kornas[4], Celine Beier[1], Martin K. Nielsen[5], Richard E. Davis[6], Jianbin Wang[6, 7], Georg von Samson-Himmelstjerna[1*]
[1]Institute for Parasitology and Tropical Veterinary Medicine, Freie Universität Berlin, Germany
[2]Institute of Biology, Molecular Parasitology, Humboldt-Universität zu Berlin, Berlin, Germany
[3]Leibniz Institute for Zoo and Wildlife Research, Research Group Ecology and Evolution of Parasite Host Interactions, Berlin, Germany
[4]Department of Zoology and Animal Welfare, University of Agriculture in Kraków, Poland
[5]Maxwell H. Gluck Equine Research Center, University of Kentucky, Lexington, USA
[6]Department of Biochemistry and Molecular Genetics, RNA Bioscience Initiative, University of Colorado School of Medicine, Aurora, USA
[7]Department of Biochemistry and Cellular and Molecular Biology, University of Tennessee, Knoxville, TN, USA

*Corresponding Author
samson.georg@fu-berlin.de

Supplementary Material

Supplementary Figure S1 Comparison of annotation of Parascaris univalens P-glycoproteins

Supplementary Figure S2 Tissue expression levels

Supplementary Figure S3 RT-PCR *Pun*Pgp-2 and *Pun*Pgp-9

Supplementary Table S1 Contig Coverage

Supplementary Table S2 Primers and sequences

Supplementary Table S3 Accession numbers

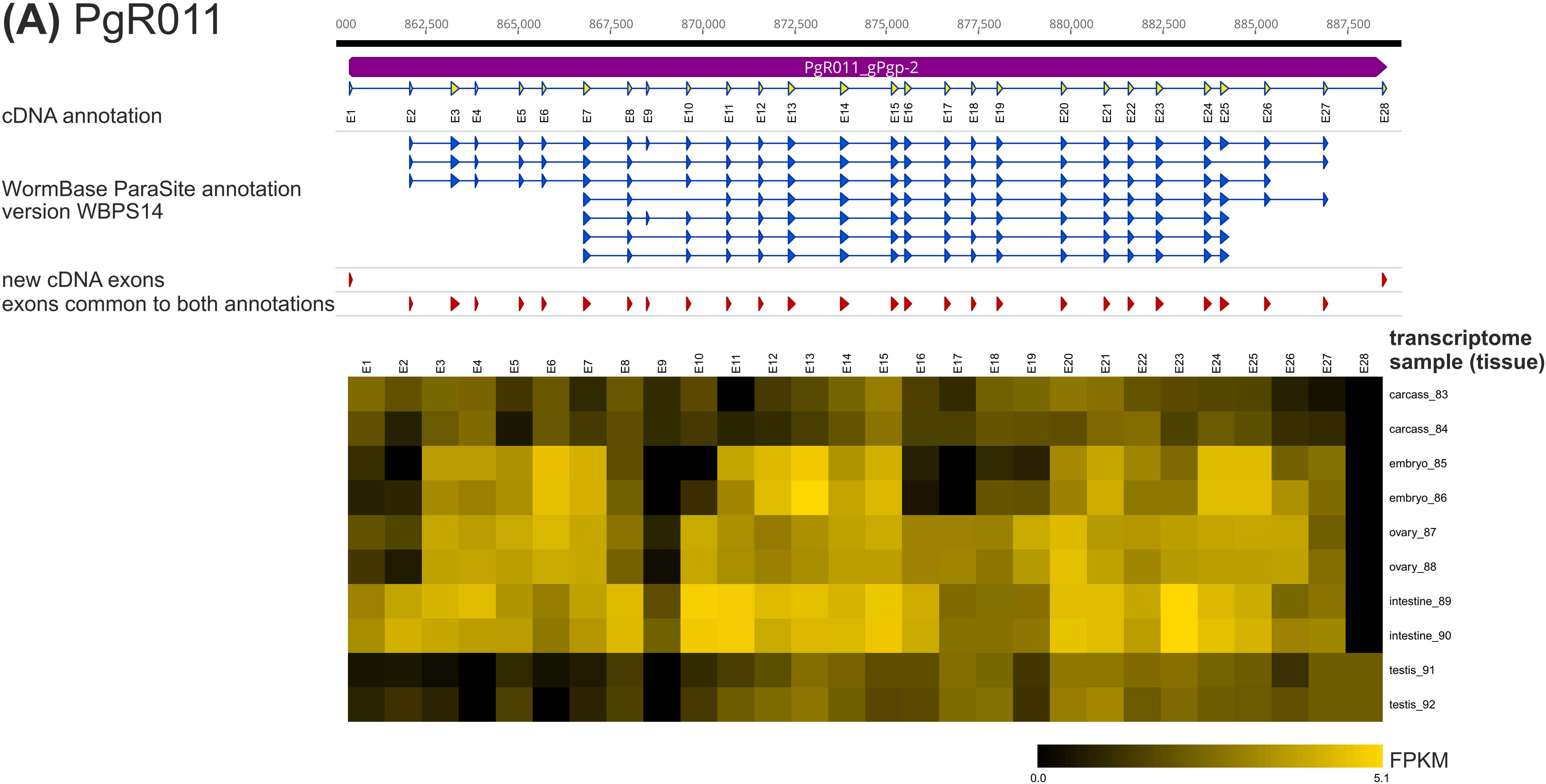

(A) PgR011
000
862,500
865,000
867,500
870,000
872,500
875,000
877,500
880,000
882,500
885,000
887,500
PgR011_gPgp-2
cDNA annotation
E1 E2 E3 E4 E5 E6 E7 E8 E9 E10 E11 E12 E13 E14 E15 E16 E17 E18 E19 E20 E21 E22 E23 E24 E25 E26 E27 E28
WormBase ParaSite annotation version WBPS14
new cDNA exons
exons common to both annotations
transcriptome sample (tissue)
carcass_83
carcass_84
embryo_85
embryo_86
ovary_87
ovary_88
intestine_89
intestine_90
testis_91
testis_92
FPKM
0.0
5.1

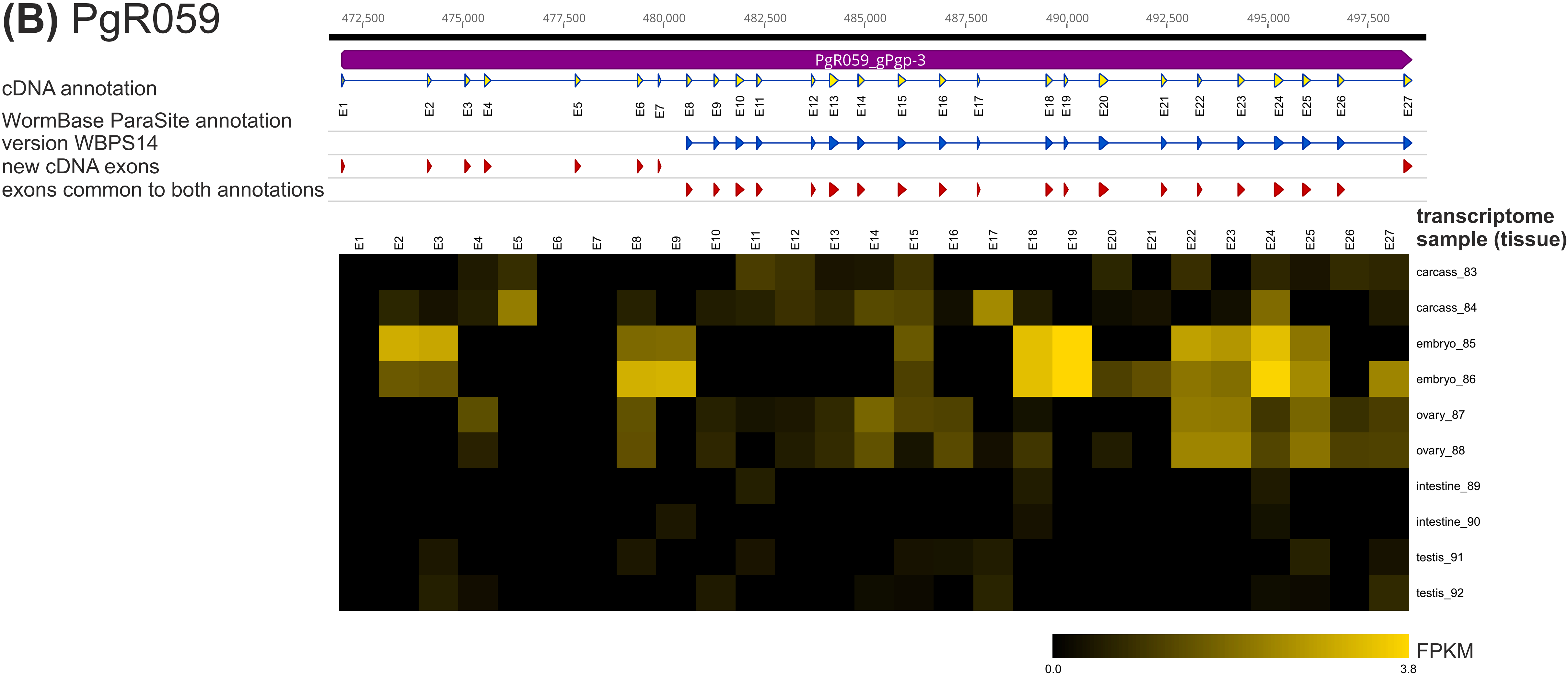
(B) PgR059
472,500
475,000
477,500
480,000
482,500
485,000
487,500
490,000
492,500
495,000
497,500
PgR059_gPgp-3
cDNA annotation
WormBase ParaSite annotation version WBPS14
new cDNA exons
exons common to both annotations
E1 E2 E3 E4 E5 E6 E7 E8 E9 E10 E11 E12 E13 E14 E15 E16 E17 E18 E19 E20 E21 E22 E23 E24 E25 E26 E27
transcriptome sample (tissue)
carcass_83
carcass_84
embryo_85
embryo_86
ovary_87
ovary_88
intestine_89
intestine_90
testis_91
testis_92
0.0
3.8
FPKM

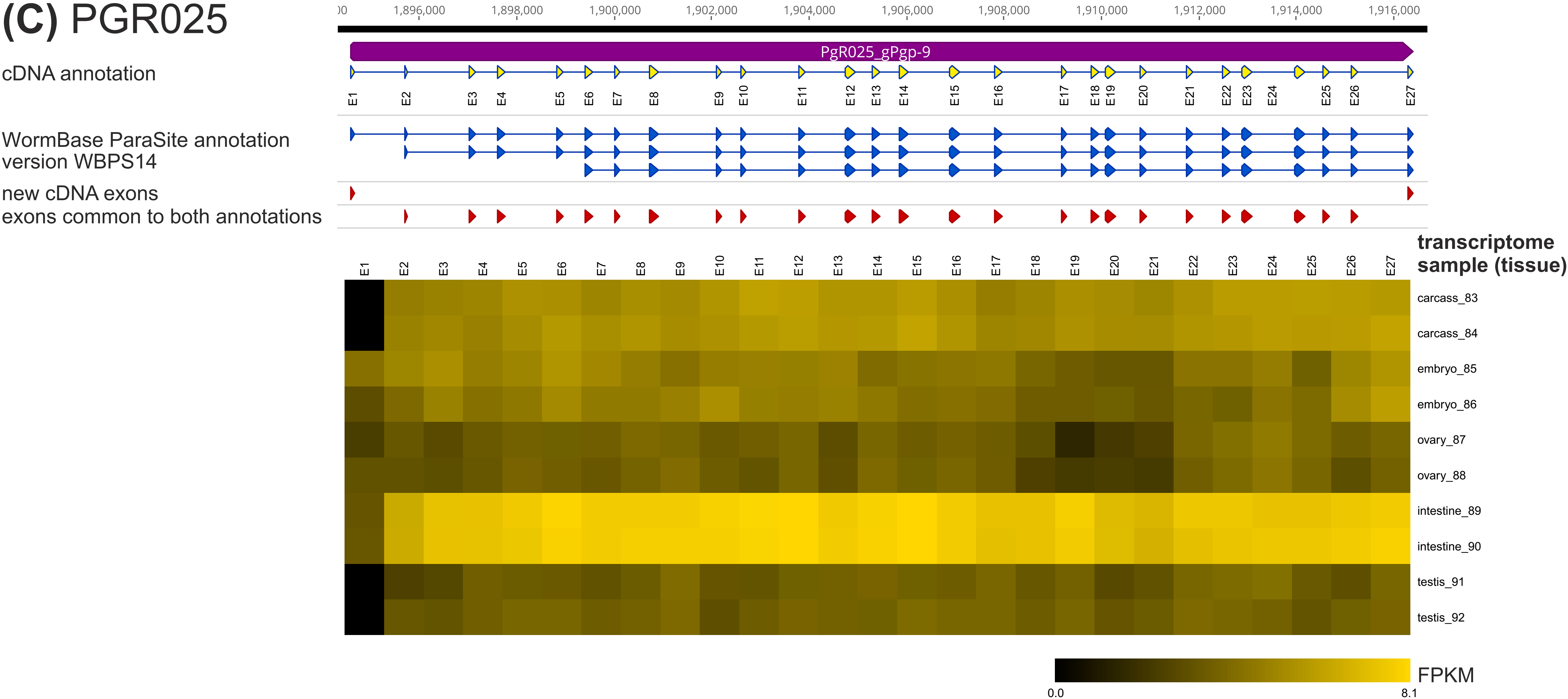
(C) PGR025
cDNA annotation
WormBase ParaSite annotation version WBPS14
new cDNA exons
exons common to both annotations
PgR025_gPgp-9
1,896,000
1,898,000
1,900,000
1,902,000
1,904,000
1,906,000
1,908,000
1,910,000
1,912,000
1,914,000
1,916,000
E1 E2 E3 E4 E5 E6 E7 E8 E9 E10 E11 E12 E13 E14 E15 E16 E17 E18 E19 E20 E21 E22 E23 E24 E25 E26 E27
transcriptome sample (tissue)
carcass_83
carcass_84
embryo_85
embryo_86
ovary_87
ovary_88
intestine_89
intestine_90
testis_91
testis_92
FPKM
0.0
8.1

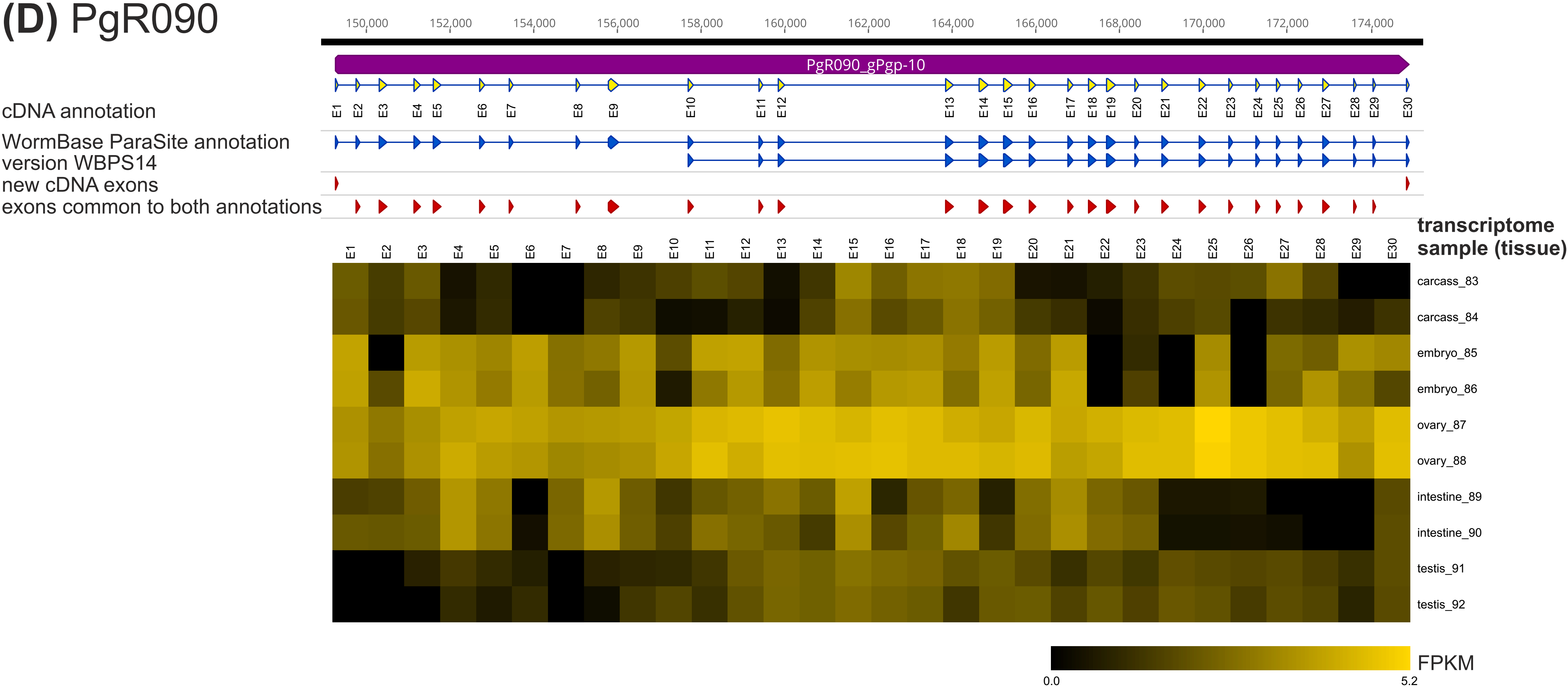

(D) PgR090
150,000
152,000
154,000
156,000
158,000
160,000
162,000
164,000
166,000
168,000
170,000
172,000
174,000
PgR090_gPgp-10
cDNA annotation
E1 E2 E3 E4 E5 E6 E7 E8 E9 E10 E11 E12 E13 E14 E15 E16 E17 E18 E19 E20 E21 E22 E23 E24 E25 E26 E27 E28 E29 E30
WormBase ParaSite annotation version WBPS14
new cDNA exons
exons common to both annotations
transcriptome sample (tissue)
E1 E2 E3 E4 E5 E6 E7 E8 E9 E10 E11 E12 E13 E14 E15 E16 E17 E18 E19 E20 E21 E22 E23 E24 E25 E26 E27 E28 E29 E30
carcass_83
carcass_84
embryo_85
embryo_86
ovary_87
ovary_88
intestine_89
intestine_90
testis_91
testis_92
FPKM
0.0
5.2

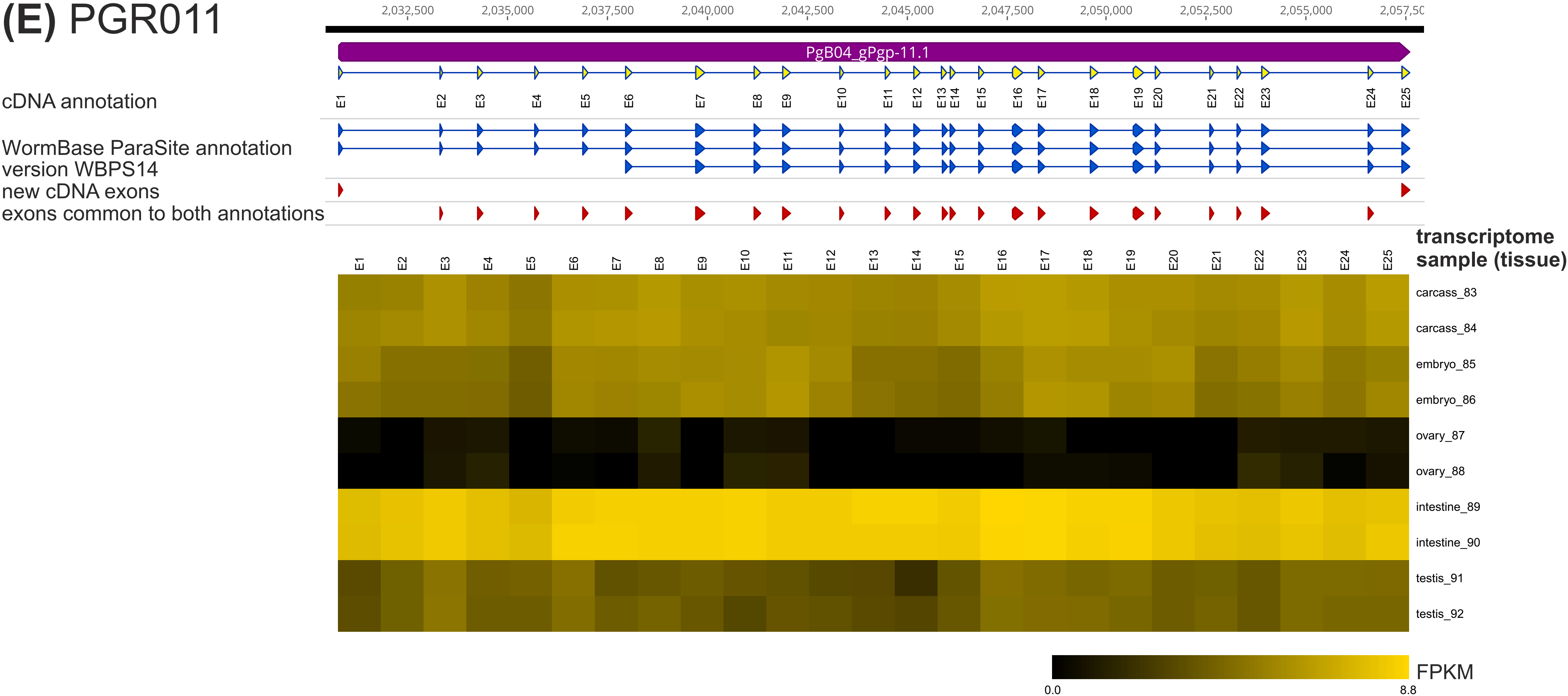

(E) PGR011
2,032,500
2,035,000
2,037,500
2,040,000
2,042,500
2,045,000
2,047,500
2,050,000
2,052,500
2,055,000
PgB04_gPgp-11.1
cDNA annotation
E1 E2 E3 E4 E5 E6 E7 E8 E9 E10 E11 E12 E13 E14 E15 E16 E17 E18 E19 E20 E21 E22 E23 E24 E25
WormBase ParaSite annotation version WBPS14
new cDNA exons
exons common to both annotations
transcriptome sample (tissue)
E1 E2 E3 E4 E5 E6 E7 E8 E9 E10 E11 E12 E13 E14 E15 E16 E17 E18 E19 E20 E21 E22 E23 E24 E25
carcass_83
carcass_84
embryo_85
embryo_86
ovary_87
ovary_88
intestine_89
intestine_90
testis_91
testis_92
FPKM
0.0
8.8

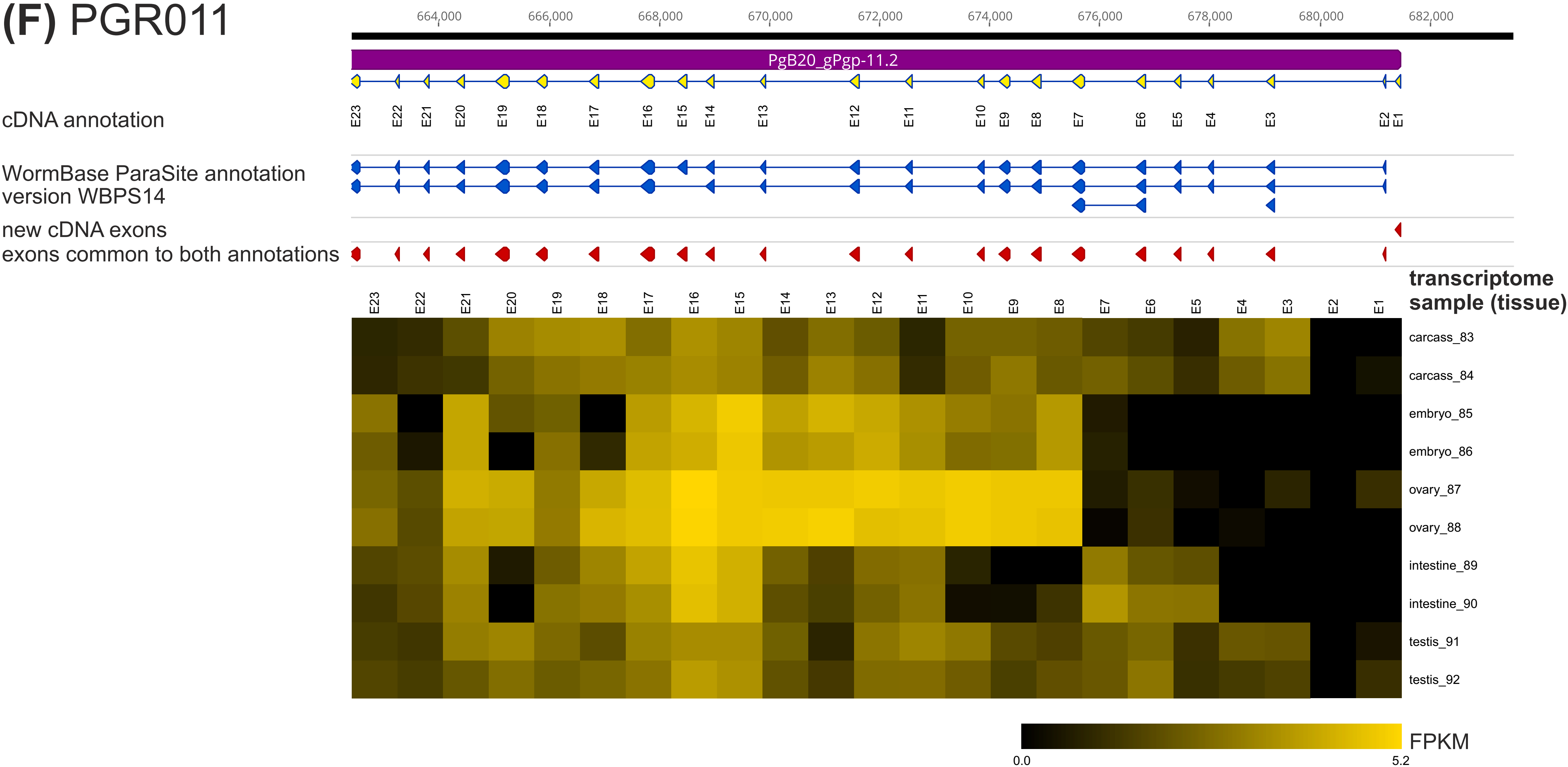

(F) PGR011
664,000
666,000
668,000
670,000
672,000
674,000
676,000
678,000
680,000
682,000
PgB20_gPgp-11.2
cDNA annotation
E23 E22 E21 E20 E19 E18 E17 E16 E15 E14 E13 E12 E11 E10 E9 E8 E7 E6 E5 E4 E3 E2 E1
WormBase ParaSite annotation
version WBPS14
new cDNA exons
exons common to both annotations
transcriptome
sample (tissue)
E23 E22 E21 E20 E19 E18 E17 E16 E15 E14 E13 E12 E11 E10 E9 E8 E7 E6 E5 E4 E3 E2 E1
carcass_83
carcass_84
embryo_85
embryo_86
ovary_87
ovary_88
intestine_89
intestine_90
testis_91
testis_92
FPKM
0.0
5.2

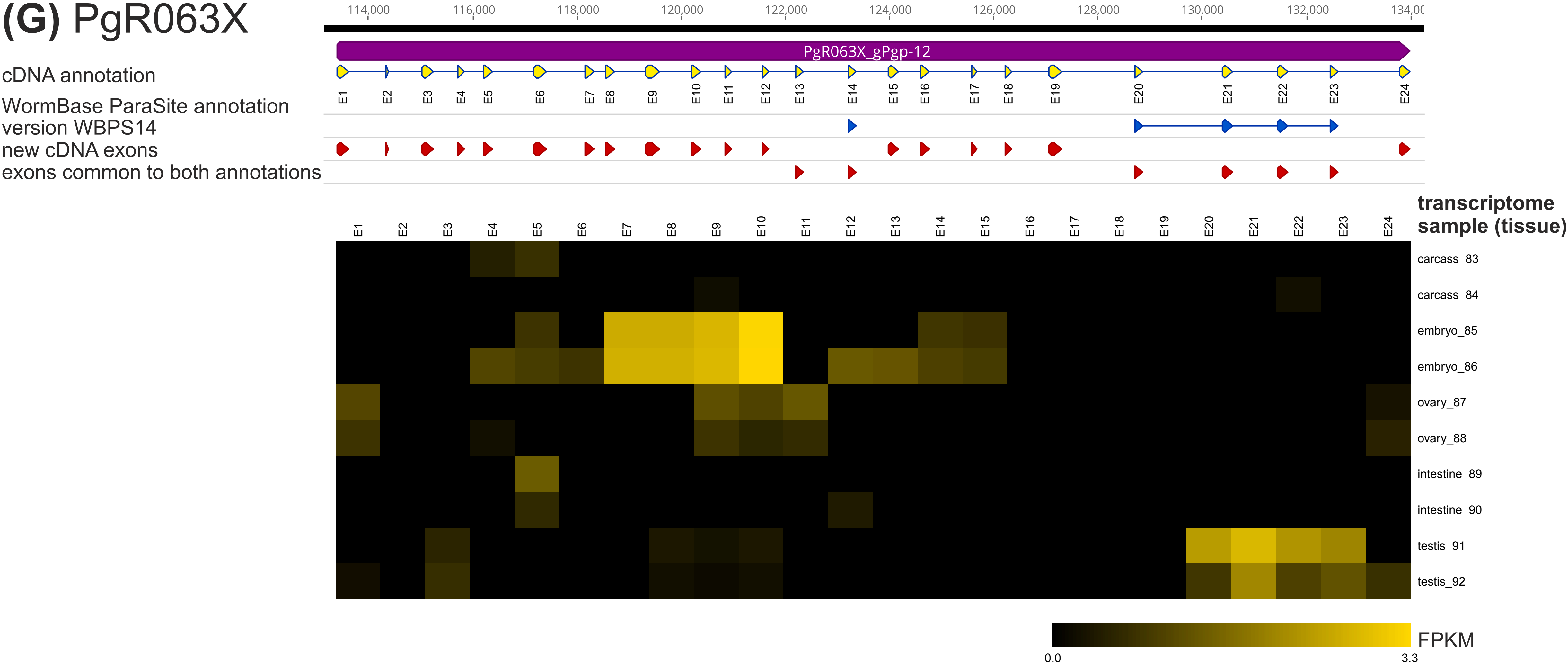
(G) PgR063X
114,000
116,000
118,000
120,000
122,000
124,000
126,000
128,000
130,000
132,000
PgR063X_gPgp-12
cDNA annotation
E1 E2 E3 E4 E5 E6 E7 E8 E9 E10 E11 E12 E13 E14 E15 E16 E17 E18 E19 E20 E21 E22 E23 E24
WormBase ParaSite annotation version WBPS14
new cDNA exons
exons common to both annotations
transcriptome sample (tissue)
carcass_83
carcass_84
embryo_85
embryo_86
ovary_87
ovary_88
intestine_89
intestine_90
testis_91
testis_92
FPKM
0.0
3.3

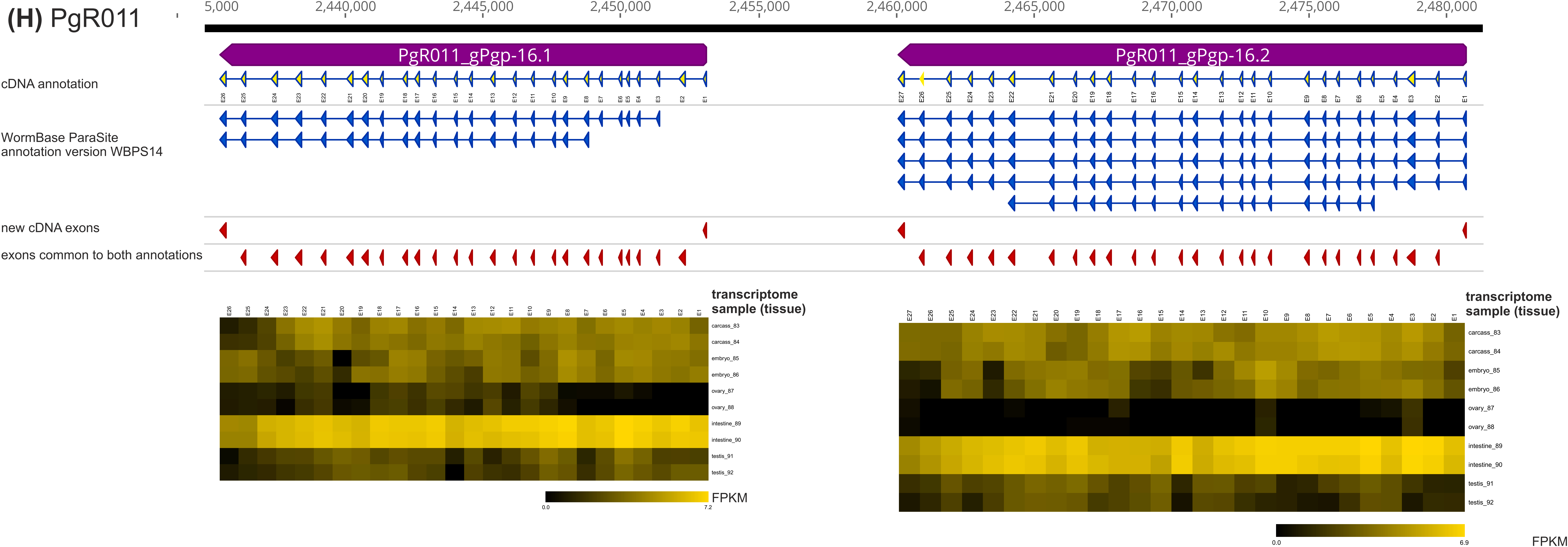

(H) PgR011
5,000
2,440,000
2,445,000
2,450,000
2,455,000
2,460,000
2,465,000
2,470,000
2,475,000
2,480,000
PgR011_gPgp-16.1
PgR011_gPgp-16.2
cDNA annotation
WormBase ParaSite annotation version WBPS14
new cDNA exons
exons common to both annotations
transcriptome sample (tissue)
carcass_83
carcass_84
embryo_85
embryo_86
ovary_87
ovary_88
intestine_89
intestine_90
testis_91
testis_92
FPKM
0.0
7.2
6.9

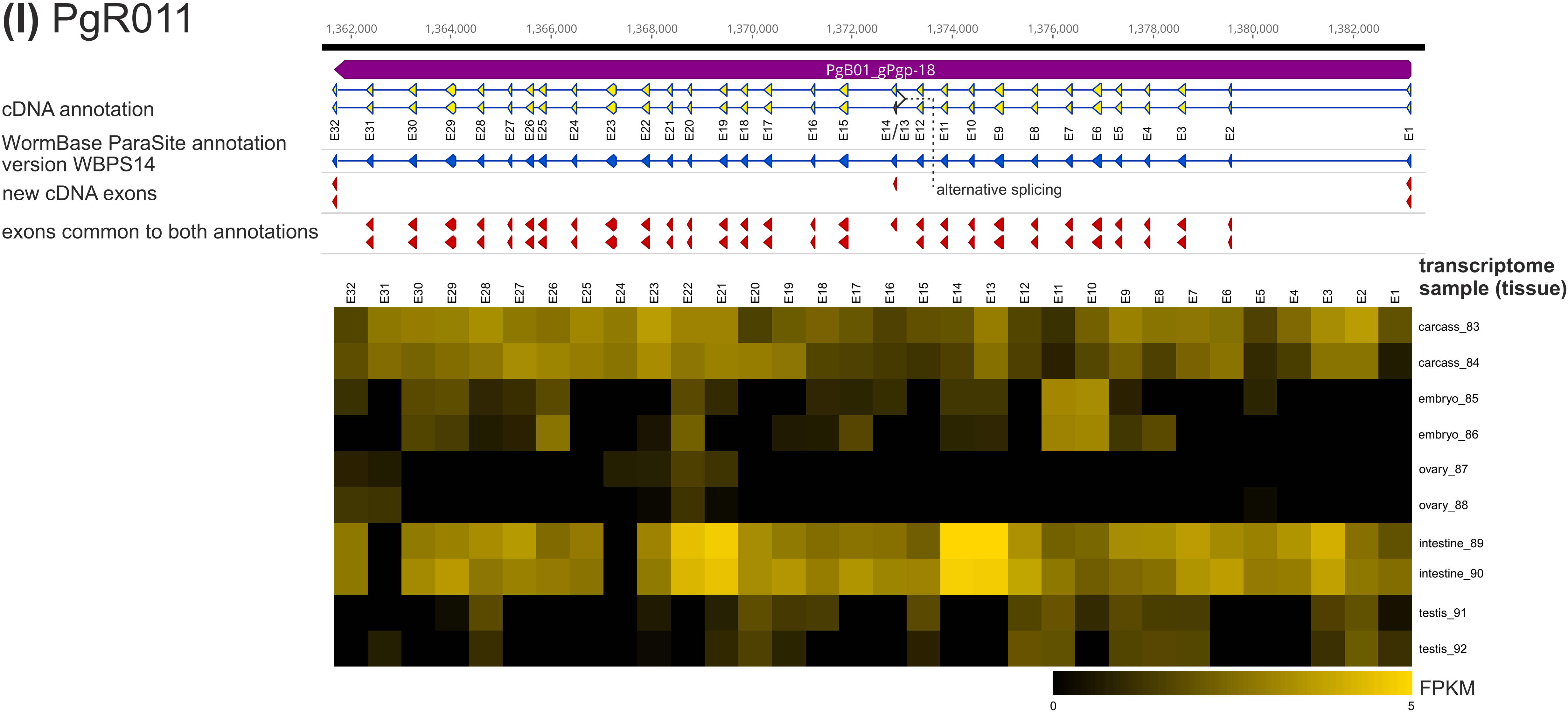
(I) PgR011
1,362,000
1,364,000
1,366,000
1,368,000
1,370,000
1,372,000
1,374,000
1,376,000
1,378,000
1,380,000
1,382,000
PgB01_gPgp-18
cDNA annotation
WormBase ParaSite annotation version WBPS14
new cDNA exons
exons common to both annotations
alternative splicing
E32 E31 E30 E29 E28 E27 E26 E25 E24 E23 E22 E21 E20 E19 E18 E17 E16 E15 E14 E13 E12 E11 E10 E9 E8 E7 E6 E5 E4 E3 E2 E1
transcriptome sample (tissue)
carcass_83
carcass_84
embryo_85
embryo_86
ovary_87
ovary_88
intestine_89
intestine_90
testis_91
testis_92
FPKM
0
5

Supplemental Figure S1 Annotation of *Parascaris univalens* Pgp

Improved annotation of experimentally verified *Parascaris univalens* Pgp exons (yellow) numbered 5’ to 3’ end were compared to original automatic annotation (WormBase ParaSite annotation version WBPS14, blue) and differences of new exons and exons common to both annotation highlighted (red). cDNA sequences of Parascaris Pgps were mapped onto the *P. univalens* genome with Splign version 2.1.0 to identify genomic coordinates of exons. Visualisation of original annotation and the newly generated annotation was made using Geneious Prime 2019.2.3. Coverage of individual exons of the new experimental cDNA exons was calculated with R package SGSeq and visualized as a heatmap (FPKM, yellow) for each tissue sample of the tissue specific transcriptome (GeoDataset accession: GSE99524). For each Pgp (A) Pgp-2, (B) Pgp-3, (C) Pgp-9, (D) Pgp-10, (E) Pgp-11.1, (F) Pgp-11.2, (G) Pgp-12, (H) Pgp-16.1 and Pgp-16.2, (I) Pgp-18 were visualized on the respective genomic locus of the respective genomic scaffold (genome assembly ASM225920v1, version WBPS14). Pgp: P-glycoprotein

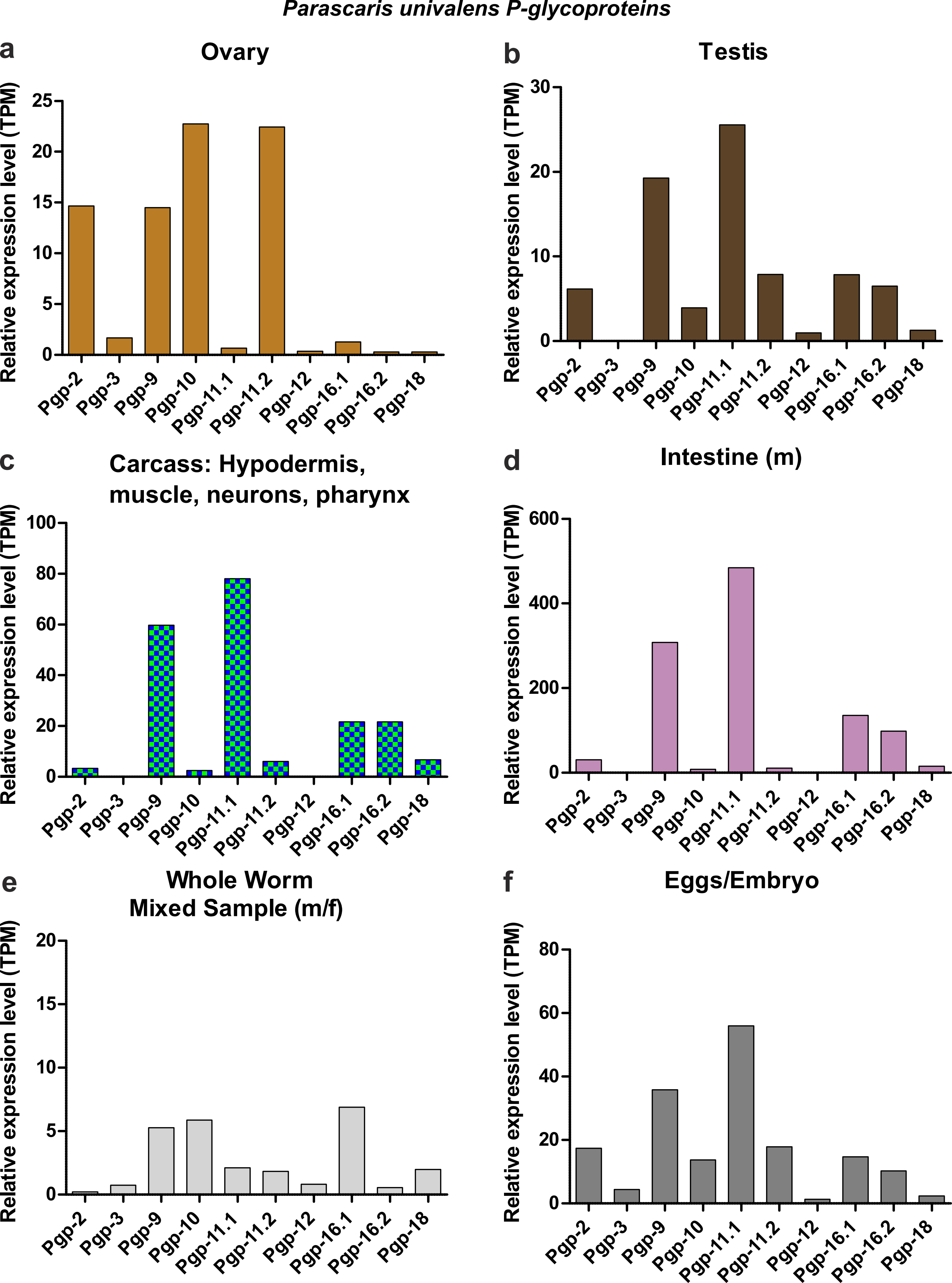
Parascaris univalens P-glycoproteins
a
Ovary
b
Testis
c
Carcass: Hypodermis, muscle, neurons, pharynx
d
Intestine (m)
e
Whole Worm Mixed Sample (m/f)
f
Eggs/Embryo
Relative expression level (TPM)
Pgp-2
Pgp-3
Pgp-9
Pgp-10
Pgp-11.1
Pgp-11.2
Pgp-12
Pgp-16.1
Pgp-16.2
Pgp-18

Supplementary Figure S2 Tissue expression levels of *Parascaris univalens* P-glycoproteins

Expression levels of *Parascaris univalens* Pgp were calculated based on transcriptome raw reads data sets (Geo Accession GSE99524, samples GSM2645460 to GSM2645464) mapped onto the the augmented (with *P. univalens* Pgp cDNA sequences) *P. univalens* genome (WormBase Parasite genome assembly ASM225920v1, version WBPS14) using STAR. Expression levels were normalised as transcripts per million (TPM) from raw read counts obtained with featureCounts. Then expression levels were visuliased by tissue of (a) the ovary (light brown), (b) the testis (brown), (c) carcass tissue including the epidermis, neurons, muscles and the pharynx (blue and green), (d) intestine (purple), (e) a whole worm sample (5'-TeloPrime library), (f) eggs. Pgp: P-glycoprotein; m: Male; f: female; TPM: transcripts per million

Supplementary Figure S3 RT-PCR of Saccharomyces cerevisiae AD1-7 expressing *Pun*Pgp-2, *Pun*Pgp-9 or lacZ

a

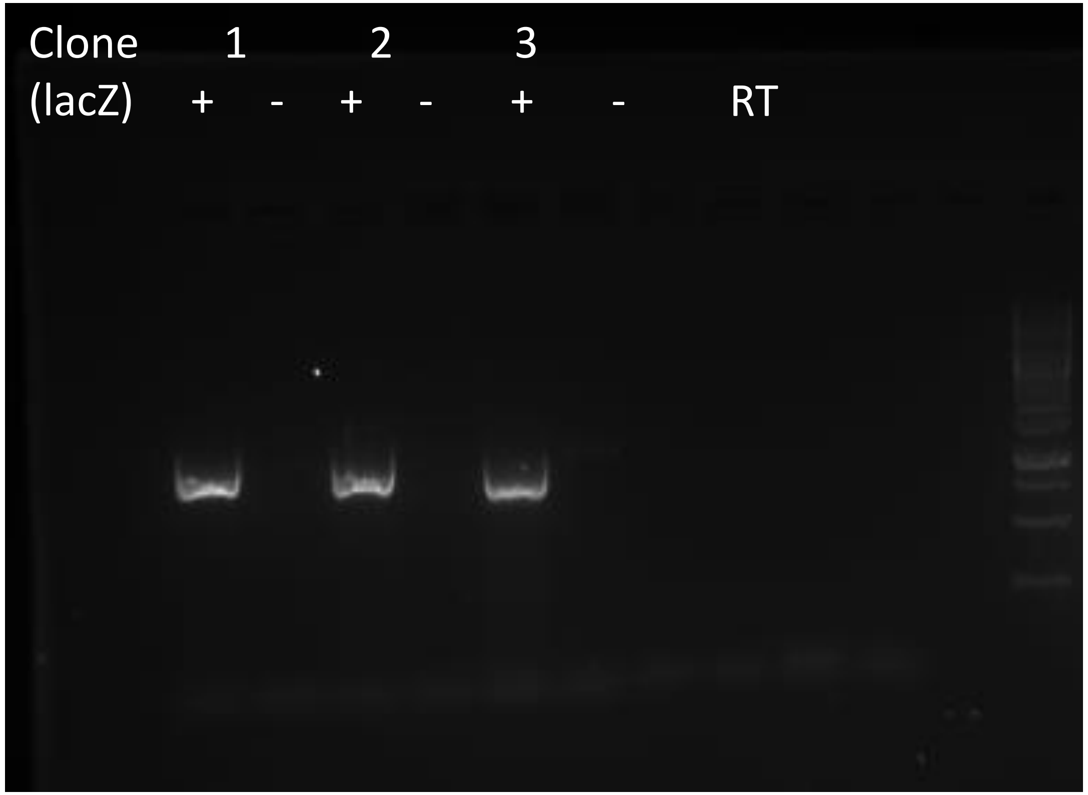

b

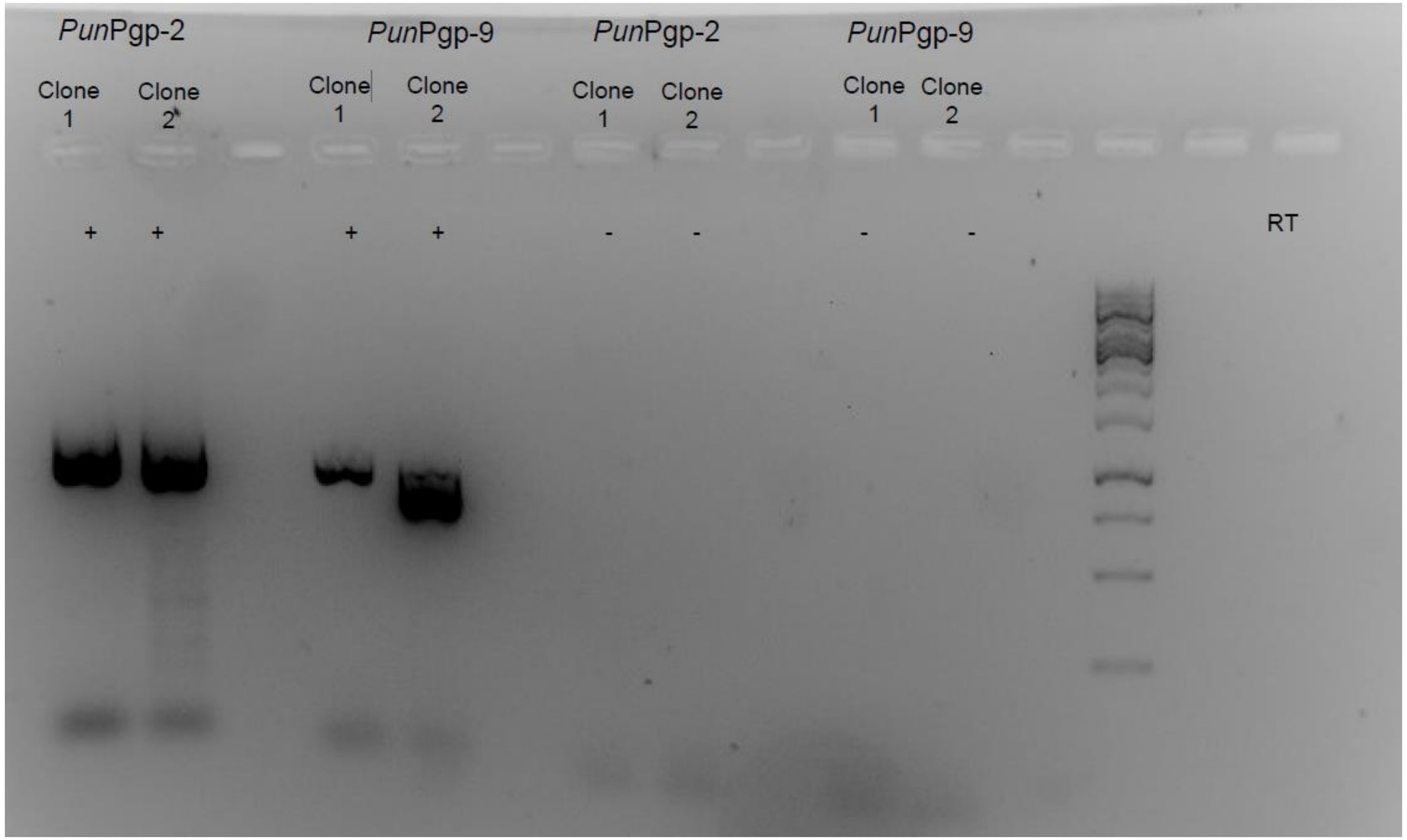

Supplementary Figure S3 RT-PCR of *Saccharomyces cerevisiae* AD1-7 expressing *Pun*Pgp-2, *Pun*Pgp-9 or lacZ
Saccharomyces cerevisiae AD1-7 expressing lacZ, *Pun*Pgp-2 or *Pun*Pgp-9 were generated through transformation. Following total RNA extraction and cDNA synthesis including a no-reverse transcriptase (RT) control, RT-PCRs were performed. PCR products were visualised with Gel-electrophoresis. Several clones were screened but only clone 1 for all genes was used for analyses. Pgp: P-glycoprotein. RT: Reverse transcriptase

Supplementary Table S1

Contig Coverage of ORFs in the tissue specific transcriptome assembly

***Pun*Pgp-2**	cDNA ORF length:	3864	Number of contigs:	7	Coverage Start:	1	Coverage End:	3864	Full ORF Coverage:	100.00%
	**Contig**	**Lenth (bp)**	**From (cDNA)**	**To (cDNA)**	**From (contig)**	**To (contig)**	**Gaps**	**Identities**	**Identities (%)**	**ORF Coverage (%)**
	hub_106215_PG_hub_106215_all_transcripts_PgR011_g057_t01	3653	1	3658	21	3653	25	3622	99.0%	94.7%
	hub_106215_PG_hub_106215_all_transcripts_PgR011_g057_t02	4339	1	3864	21	3857	27	3825	99.0%	100.0%
	hub_106215_PG_hub_106215_all_transcripts_PgR011_g057_t03	3833	1	3841	21	3833	28	3803	99.7%	99.4%
	hub_106215_PG_hub_106215_all_transcripts_PgR011_g057_t04	3638	407	3583	114	3269	21	3148	99.1%	82.2%
			3376	3511	3271	3406	0	136	100.0%	3.5%
	hub_106215_PG_hub_106215_all_transcripts_PgR011_g057_t05	3552	407	3583	114	3269	21	3148	99.1%	82.2%
			3376	3658	3271	3552	1	281	99.3%	7.3%
	hub_106215_PG_hub_106215_all_transcripts_PgR011_g057_t06	4009	407	3864	114	3527	44	3404	99.7%	89.5%
	hub_106215_PG_hub_106215_all_transcripts_PgR011_g057_t07	3525	407	3583	1	3156	21	3148	99.1%	82.2%
			3376	3511	3158	3293	0	136	100.0%	3.5%
***Pun*Pgp-3**	cDNA ORF length:	3723	Number of contigs:	5	Coverage Start:	591	Coverage End:	3971	Overall ORF Coverage (%)	90.8%
	**Contig**	**Lenth (bp)**	**From (cDNA)**	**To (cDNA)**	**From (contig)**	**To (contig)**	**Gaps**	**Identities**	**Identities (%)**	**ORF Coverage (%)**
	hub_106215_PG_hub_106215_all_transcripts_PgR059_g034_t01	3248	2113	3723	1604	3205	9	1602	100.0%	43.3%
			591	2112	1	1512	10	1509	99.8%	40.9%
***Pun*Pgp-9**	cDNA ORF length:	3948	Number of contigs:	3	Coverage Start:	1	Coverage End:	3948	Full ORF Coverage:	100.00%
	**Contig**	**Lenth (bp)**	**From (cDNA)**	**To (cDNA)**	**From (contig)**	**To (contig)**	**Gaps**	**Identities**	**Identities (%)**	**ORF Coverage (%)**
	hub_106215_PG_hub_106215_all_transcripts_PgR025_g103_t03	5506	1	3948	793	4714	26	3922	99.3%	100.0%
	hub_106215_PG_hub_106215_all_transcripts_PgR025_g103_t02	5620	76	3948	981	4828	25	3848	99.4%	98.1%
	hub_106215_PG_hub_106215_all_transcripts_PgR025_g103_t01	6823	276	3948	2382	6031	23	3650	100.0%	93.0%
			76	278	1749	1950	1	202	100.0%	5.1%
***Pun*Pgp-10**	cDNA ORF length:	3924	Number of contigs:	6	Coverage Start:	1	Coverage End:	3924	Overall ORF Coverage (%)	100.00%
	**Contig**	**Lenth (bp)**	**From (cDNA)**	**To (cDNA)**	**From (contig)**	**To (contig)**	**Gaps**	**Identities**	**Identities (%)**	**ORF Coverage (%)**
	hub_106215_PG_hub_106215_all_transcripts_PgR090_g012_t01	4387	1	3924	266	4160	29	3888	99.8%	100.0%
	hub_106215_PG_hub_106215_all_transcripts_PgR090_g012_t02	2987	1144	3924	1	2760	21	2757	99.9%	70.9%
***Pun*Pgp-11.1**	cDNA ORF length:	3858	Number of contigs:	3	Coverage Start:	1	Coverage End:	3858	Full ORF Coverage:	100.00%
	**Contig**	**Lenth (bp)**	**From (cDNA)**	**To (cDNA)**	**From (contig)**	**To (contig)**	**Gaps**	**Identities**	**Identities (%)**	**ORF Coverage (%)**
	hub_106215_PG_hub_106215_all_transcripts_PgB04_g139_t01	5657	1	3858	1574	5430	24	3825	99.1%	100.0%
	hub_106215_PG_hub_106215_all_transcripts_PgB04_g139_t02	4968	1	3858	885	4718	24	3825	99.8%	100.0%
	hub_106215_PG_hub_106215_all_transcripts_PgB04_g139_t03	4359	388	3858	659	4109	20	3442	99.7%	90.0%
***Pun*Pgp-11.2**	cDNA ORF length:	3909	Number of contigs:	4	Coverage Start:	1	Coverage End:	3909	Overall ORF Coverage (%)	100.00%
	**Contig**	**Lenth (bp)**	**From (cDNA)**	**To (cDNA)**	**From (contig)**	**To (contig)**	**Gaps**	**Identities**	**Identities (%)**	**ORF Coverage (%)**
	hub_106215_PG_hub_106215_all_transcripts_PgB20_g052_t01	4103	1	3909	5	3889	24	3876	99.8%	100.0%
	hub_106215_PG_hub_106215_all_transcripts_PgB20_g052_t02	4097	1	3909	5	3883	30	3870	99.8%	100.0%
	hub_106215_PG_hub_106215_all_transcripts_PgB20_g052_t03	620	448	1070	1	620	3	618	99.7%	15.9%
	hub_106215_PG_hub_106215_all_transcripts_PgB20_g052_t04	338	166	386	119	338	1	219	99.5%	5.7%
***Pun*Pgp-12**	cDNA ORF length:	4056	Number of contigs:	3	Coverage Start:	1464	Coverage End:	3971	Overall ORF Coverage (%)	61.83%

	**Contig**	**Lenth (bp)**	**From (cDNA)**	**To (cDNA)**	**From (contig)**	**To (contig)**	**Gaps**	**Identities**	**Identities (%)**	**ORF Coverage (%)**
	hub_106215_PG_hub_106215_all_transcripts_PgR063X_g008_t01	1038	2929	3971	1	1038	5	1037	99.9%	28.0%
	hub_106215_PG_hub_106215_all_transcripts_PgR063X_g007_t01	650	1917	2418	152	650	3	499	100.0%	13.5%
	hub_106215_PG_hub_106215_all_transcripts_PgR063X_g006_t01	264	1464	1728	1	264	1	264	100.0%	7.1%
***Pun*Pgp-16.1**	cDNA ORF length:	3855	Number of contigs:	2	Coverage Start:	84	Coverage End:	3855	Overall ORF Coverage (%)	97.85%
	**Contig**	**Lenth (bp)**	**From (cDNA)**	**To (cDNA)**	**From (contig)**	**To (contig)**	**Gaps**	**Identities**	**Identities (%)**	**ORF Coverage (%)**
	hub_106215_PG_hub_106215_all_transcripts_PgR011_g140_t01	5610	84	833	1165	1909	5	744	99.2%	19.5%
			829	3855	2490	5497	19	3004	99.2%	78.5%
	hub_106215_PG_hub_106215_all_transcripts_PgR011_g140_t02	5024	84	3855	1165	4911	25	3742	99.9%	97.8%
***Pun*Pgp-16.2**	cDNA ORF length:	3864	Number of contigs:	5	Coverage Start:	1	Coverage End:	3864	Overall ORF Coverage (%)	100.00%
	**Contig**	**Lenth (bp)**	**From (cDNA)**	**To (cDNA)**	**From (contig)**	**To (contig)**	**Gaps**	**Identities**	**Identities (%)**	**ORF Coverage (%)**
	hub_106215_PG_hub_106215_all_transcripts_PgR011_g141_t01	5174	1	3864	625	4462	26	3835	99.2%	100.0%
	hub_106215_PG_hub_106215_all_transcripts_PgR011_g141_t02	5170	1	3864	621	4458	26	3835	99.2%	100.0%
	hub_106215_PG_hub_106215_all_transcripts_PgR011_g141_t03	6530	1	3864	1981	5818	26	3835	99.9%	100.0%
	hub_106215_PG_hub_106215_all_transcripts_PgR011_g141_t04	7109	1	3864	2560	6397	26	3835	99.9%	100.0%
	hub_106215_PG_hub_106215_all_transcripts_PgR011_g141_t05	3529	411	3124	810	3505	20	2692	99.9%	70.2%
***Pun*Pgp-18**	cDNA ORF length:	3888	Number of contigs:	1	Coverage Start:	1	Coverage End:	3888	Overall ORF Coverage (%)	100.00%
	**Contig**	**Lenth (bp)**	**From (cDNA)**	**To (cDNA)**	**From (contig)**	**To (contig)**	**Gaps**	**Identities**	**Identities (%)**	**ORF Coverage (%)**
	hub_106215_PG_hub_106215_all_transcripts_PgB01_g089_t01	4070	1	3888	26	3892	37	3858	99.8%	100.0%

Supplementary Table S1. Contigs encoding P-glycoproteins of the tissue specific transcriptome (GEO accession number: GSE99524). For each P-glycoprotein ORF and for each contig length, ORF coordinates (from/to cDNA) as well as the corresponding contig coordinates (from/to contig), gaps, identities, identity percentage and ORF coverage (ignoring gaps) is listed. Number of contigs vary strongly between Pgp and do not correspond to their expression level. Complete ORF coverage was not available for several Pgp. Pgp: P-glycoprotein

Supplementary Table S2

a) Full Length P-glycoprotein Open Reading Frame Primers

Gene	Direction	contig of origin	Sequence (5'-3')	Primer length	Tm (°C)	Fragment Size (bp)
*Pun*Pgp-2	Forward	hub_106215_PG_hub_106215_all_transcripts_PgR011_g057_t02	ACTGGTGGTTATCAACGTG	19	71.4	4065
	Reverse	hub_106215_PG_hub_106215_all_transcripts_PgR011_g057_t02	ATCCACACTACAAAACGTGA	20		
*Pun*Pgp-10	Forward	hub_106215_PG_hub_106215_all_transcripts_PgR090_g012_t01	CAACTTGTTCGGCAGCTTCAGTTTACCGACAA	32	55.0	4547
	Reverse	hub_106215_PG_hub_106215_all_transcripts_PgR090_g012_t01	GAGAAGTGGTAGAACAAAGGATGAGGTGATAA	32		
*Pun*Pgp-3	Forward	comp75122_c0_seq1	TGAGAGATGTCTTCACGAAGAAAGG	25	55.0	4091
	Reverse	comp75122_c0_seq1	CAACTTAAAAGATCTTGTTTCCTGA	25		
*Pun*Pgp-18	Forward	hub_106215_PG_hub_106215_all_transcripts_PgB01_g089_t01	CTATCAGGTAAAGTGAATCCAAGAAATGGGT	31	65.0	4120
	Reverse	hub_106215_PG_hub_106215_all_transcripts_PgB01_g089_t01	ATTGATTAAGAGATGAAATGAATGATAGCGAA	32		
*Pun*Pgp-11.2	Forward	hub_106215_PG_hub_106215_all_transcripts_PgB20_g052_t01	AAAATGACCGACAACGACAAGTCAAAATGCAG	32	60.0	4105

	Reverse	hub_106215_PG_hub_106215_all_transcripts_PgB20_g052_t01	TTTCCTTGTTAGATATAACGAAACGCTCATGG	32		
*Pun*Pgp-3	Forward	comp75122_c0_seq1	ATGGCACGATTCATGTCAAGGTTCTATC	28	55.0	3765
	Reverse	comp75122_c0_seq1	GATGGTTTCTGTGATATATAGCGTTTCTCTCT	32		
*Pun*Pgp-16.2	Forward	hub_106215_PG_hub_106215_all_transcripts_PgR011_g141_t01	GCTACTAATCGTCTAGCTCTATTGGTCATCAA	32	60.0	4332
	Reverse	hub_106215_PG_hub_106215_all_transcripts_PgR011_g141_t01	TTTCTAGGCCTGCTGCTATTCCTTTATTGCAA	32		
*Pun*Pgp-9	Forward	hub_106215_PG_hub_106215_all_transcripts_PgB04_g139_t01	TCTCTCGTCGATGTTCACACGTT	23	59.0	4110
	Reverse	hub_106215_PG_hub_106215_all_transcripts_PgB04_g139_t01	TGGTGATTATCGCAGCAACAGG	22		

b) Additional Primers

Gene	Direction	contig of origin	Sequence (5'-3')	Primer length	Tm (°C)	Fragment Size (bp)	Purpose
*Pun*Pgp-12	Forward	hub_106215_PG_hub_106215_all_transcripts_PgR063X_g008_t01	TCAACTTCTAAGCAGAGAGGCACG	24	65	634	Verification of fragment
	Reverse	hub_106215_PG_hub_106215_all_transcripts_PgR063X_g008_t01	ATCGTGGTTCTTGTCCCACCAATG	24			

	Forward	hub_106215_PG_hub_106215_all_transcripts_PgR063X_g008_t01	AGGTCTCGACACAGAAG TTGGAGA	24	62	226	
	Reverse	hub_106215_PG_hub_106215_all_transcripts_PgR063X_g008_t01	TGCTGAATTG ACGATAGTCG ATGTGC	28			
	Forward	comp29735_c0_seq1	TCTTcACGAAGAAAGGT TCAAGATGAGCA	29	65	421	
	Reverse	comp29735_c0_seq1	GATAGGCGGCAAGGAG AAATTCG	23			
	Forward	comp29735_c0_seq1	CGGCAGGTTTCATAACA ATTTATCG	25	65	270	
	Reverse	comp29735_c0_seq1	TAACATACGAGCGTCGA TTACCGC	24			
	Forward	hub_106215_PG_hub_106215_all_transcripts_PgR063X_g008_t01	TCAACTTCTAAGCAGAG AGGCACG	24	65		Verification of g008/29735 as one Pgp
	Reverse	comp29735_c0_seq1	TAACATACGAGCGTCGA TTACCGC	24			
	Forward	Splice Leader (SL) *Ascaris suum*	GGTTTAATTACCCAAGT TTGAG	22			Amplification of 5' end
	Reverse	Sequencing product of PgR063X_g008_t01	AGTGATACTGGTTTTGC CTCG	21	60	unspecific	Reverse for SL
	Reverse 2	Sequencing product of PgR063X_g008_t01	TTGTTTCGCCAAATCAA GCC	20	60	~220bp	Nested SL reverse
	Forward	Sequencing product of comp29735_c0_seq1	GTGCGTTTCGATGGACG TGAC	22	70		3'RACE
	Forward	Sequencing product of comp29735_c0_seq1	AGAAATCAGATGGCATT GGCGG	23		700bp	nested_1 (3'RACE)
	Forward	Sequencing product of3' RACE SP6	GGGTCTCGACACAGAA GTTGGAGA	24		600bp	nested_2 (3'RACE)

c) Full Length P-glycoprotein Sequencing Primers

Gene	Forward Primers	Sequence (5'-3')	Reverse Primers	Sequence (5'-3')
*Pun*Pgp-2	M13-24F-BLUE	GTAAAACGACGGCCAGTGAGCGCG	M13-24R-BLUE	AACAGCTATGACCATGATTACGCC
	F1	CGTCAGAAATATCTGAAAGCAATA	R1	GTGTATGTTGGCTAATTTGGCG
	F2	GCTGGATCGAGAAGTTTCACAC	R2	CATCAGTGAGACGAGCGGTAAG
	F3	TATTCGATGGAACCATCGAGAGTA	R3	TCCCGGTGGAATGTCTTTGATCAT
	F4	TTGTGCACTCGATTTGCGACCGAT	R4	CGGTTCTTGCGATACGATTCCGAT
	F5	GTGCAGCAATCGTCAAAGAACT		
*Pun*Pgp-10	M13-24F-BLUE	GTAAAACGACGGCCAGTGAGCGCG	M13-24R-BLUE	AACAGCTATGACCATGATTACGCC
	F1	TCTCGTTGGCGGTTTGTTTC	R1	AGGTAAAATCGGCCTTGAAGG
	F2	TCCAATGGGCGTTTAATGCT G	R2	ACATCACCCAGTGTCACATC
	F3	TCTGGTGGTCAAAAGCAACG	R3	ACCGGAAGAGCTATTCCAAG
	F4	TCGTTACCCGGTAAGGATAC	R4	ACGTATAACAGCTCGGGCGA
*Pun*Pgp-18	M13-24F-BLUE	ACGATGATCGATCGATACTCG	M13-24R-BLUE	AACAGCTATGACCATGATTACGCC

	F1	TCGATTCTCACAGGTGCAAC	R1	TGCGAAGTCCATTGCTACTG
	F2	ATGGGCGAGTGGTAGAGACG	R2	AGTGACATCTGCCAACCGAAG
	F3	ATGTCACTCGTTACTCTTGTAA	R3	AGATCGACTGTCGTCCATGT
	F4	GATGACGATCCAGATGACGGG	R4	CAGGTTCTATCTCACCTGAGC
*Pun*Pgp-12	M13-24F-BLUE	ACGATGATCGATCGATACTCG	M13-24R-BLUE	AACAGCTATGACCATGATTACGCC
	F1	AGGTGCTATTGCCGAGGAATC	R1	TAGCCCAGCAGCAGTTCTTG
	F2	TCCTGAGGGATATCAGACTC	R2	ATATGCACCCGCAAGTGCTC
	F3	ATGTCTTTCGAGCACTTGCG	R3	GTCTGATATCCCTCAGGAAG
	F4	CAAGAACTGCTGCTGGGCTA	R4	ATTCCTCGGCAATAGCACCTG
*Pun*Pgp-11.2	M13-24F-BLUE	ACGATGATCGATCGATACTCG	M13-24R-BLUE	AACAGCTATGACCATGATTACGCC
	F1	TGCTACAGGC GATATTGACG	R1	ATCGCCTGTAGCACTCTCTG
	F2	TGCTACAGGC GATATTGACG	R2	TCGAGTATCGATCGATCATCG
	F3	TACTGGATGAAGCGACTAGC	R3	TGCTCTGGGTTCTCTTGTTC
	F4	TTCACTTTCGGCATCGAGC	R4	AGAGAACCCAGAGCATGGAA C
*Pun*Pgp-3	M13-24F-BLUE	ACGATGATCGATCGATACTCG	M13-24R-BLUE	AACAGCTATGACCATGATTACGCC

	F1	TGAAGAAGGCTCGTAAGATGGG	R1	AGCACCGGTAAATATTCCCGTC
	F2	TTCAAAAGGCTCTTGACCAGGC	R2	TCGCTTTCTGTGTCCAACGCAC
	F3	TGATAGCAGGAGTTGGAATCGC	R3	GATAGCAGGAGTTGGAATCGCA
	F4	TATCTGAAGCGGGTCTACGTCAG	R4	TTGTCGTCGATTGGGATAAGCG
*Pun*Pgp-16.2	M13-24F-BLUE	ACGATGATCGATCGATACTCG	M13-24R-BLUE	AACAGCTATGACCATGATTACGCC
	F1	ACATCTCGTGAAGCGACGTTG	R1	GTACAAGGTCAGCTCGATTG
	F2	CAATCGAGCTGACCTTGTAC	R2	TCTTCCAACGTCGCTTCACG A
	F3	GATTGCAAGACGTCCATCAG	R2-1	TTGGTGACAAACTGGGCAAG
	F4	TAGTTCCTCTGATGCAGGAC	R3	CCACTTATAGTCGTCGCAAC
	F5	GATGCGGCTATTTTGTCGAG	R4	GTGTCTGTCG CATTCTTCTT C
	F6	AAGTGGCCAATAAACCGGCAC	R5	TCATCGAGCGTTCCGAATTG
	F7	TCGCATTCGCCTATTGTGCAG	R6	TGGTGCACAATTTCCGCAAC
*Pun*Pgp-9	M13-24F-BLUE	GTAAAACGACGGCCAGTGAGCGCG	M13-24R-BLUE	AACAGCTATGACCATGATTACGCC
	F1	GAGTCAGTTCTTTGCCGGATTT	R1	GTGAGAGTGACATCATAATCAA
	F2	TTGGACCAAGTGGATGTGGTAA	R2	ACAGTTCCATCAATTGGGTCAT

	F3	GGAAACGAGGATGTCACTGAG	R3	CGCTCCTACGACAAGAAACATG
	F4	GATCAGATGC TCAGCAATGG TC	R4	CTCAGTGACATCCTCGTTTC C
	F5	TTCGCATATCCAGAACGACCAG		

d) Codon optimized constructs for expression in *Saccharomyces cerevisiae* of *Pun*Pgp-2 and *Pun*Pgp-9
>*Pun*Pgp-2_codon_optimized
ATGATGCGTTCAAGTCAGGATAATGAAAAAGTTCCGCTGATTTCAGCCAGTATTGCGTCATACGAGTCTTTTAACTCA
AGCAATTGCAAGCGTCCTTCATCCCTTAAGGTCAAGGAGAAAGGGCTTTTCTCATACACGACGTGTCTAGACTATTG
TCTAATCCTTATGGGCACCTTCGCAGGTATAGCACACGGAACCGGCTTTCCTCTTTTAAGTATAGTGCTAGGGGGGA
TGACAACGATATTCCTGAGAGCCGAGAACTCCGACTTTGTTCGTGGTTATTCTGTTATAGTCAACAATTCAGCCCTTA
GCCAGATTACGAAGGAAGAGTTCGACGCTTCTGTTACCACCTATTGCCTTTACTACCTATTGATAGGCGTATTTATGT
TTATATCCAGCTATATACAGATCGCTTGCTGGGAGTCTTTTAGTGAGAGAACCACTCATAGGATAAGACAAAAATATT
TAAAAGCGATACTGAGGCAGCAAATTGCCTGGTTCGATACCCAACAGAGTGGCAATCTTACGGCCCGTTTGACTGA
CGACCTGGAACGTGTTAGAGAGGGGTTAGGCGATAAGCTAAGCATGATGATTCAACTGATGGCCGCCTTCATCGCG
GGATTTATAGTCGGCTTTATATATAATTGGCGTATGACTCTAGTGATGATGGCTTTCGCCCCATTGAACGCCCTGACA
GGCGCATGGATGTCTCGTATGGCGGCGACGAGAACACAGGTCGAGCAAGAGAAGTATGCTGTGGCCGGGGCTATA
GCAGAAGAAACCTTTAGTAGCATAAGAACCGTTCATAGTCTGAACGGAGCAACCAGAGAAATCGCAAGATATGAGAA
GGCCTTAGAGGATGGGAGGCGTACTGGGCGTTTGAAATACTTATATCTTGGGACCGGAATGGCGTTGAATTACCTT
ATTGTGTATGCCAGCTACGCAGTGGCCTTTTGGTACGGCAGTCTGATCATAATCGGTGATCCGACTTTCGACAGAGG
CTCCGTGTTCACGGTATTCTTTAGTGTGATGAGTGGAAGCATGGCGCTAGGTGGTGCCCTTCCAAACATGGCTACTT
TTGCTATGGCGAGAGGTGCAGCAAGAAAAGTACTGAGTGTCATCAACAGTGTGCCTATTATTGACCCTTACTCCAGC
AGTGGTACATTTCCCTCAAAGCTAAAGGGGGCGATCAGTTTTCAAAATGTGTCCTTTTCCTATCCGATCCGTAAAGAC
ATTCAAATTTTGGACCGTATTACTTTTGATATTAGTCCCGGTAGAAAAATCGCGTTGGTGGGAAGCAGTGGTTGTGG
GAAATCTACCGTGATGCACCTGCTTTTGAGGTTTTATGATCCGGACCTTGGTATGGTGACTCTTGATGGTTATGACAT
TCGTAGTCTTAATGTCCGTCGTCTGAGGGATGCAATAGGGATAGTGAGCCAAGAGCCGATACTGTTTGATGGCACG
ATAGAATCAAATATCCGTCTTGGGTGGGAGAAAGCTACGAGAGAGGACATTGTCAGGGCATGTAAACAAGCTAACG
CTTGGGAATTTATTCAGCTTCTGCCCGACGGGCTTAGTACCAGAGTAGGCGAGCGTGGCGTGCAACTATCAGGGGG
CCAGAAACAAAGAATTGCCATTGCACGTGCACTAATAAAGGATCCCCAAATTTTGCTTCTGGACGAGGCAACCAGCG
CACTTGACACGGAGAGCGAATCCGTAGTACAAAAGGCTCTTGAACAGGCTCAGATCGGTAGAACTACAATCACGATT
GCACACCGTCTGTCAACTATCAGGGACGTGGATGAGATCCTAGTCTTTAAAAACGGATCCATCGTCGAAAGGGGAA
CGCACATAGAGTTAATTGCCGCGCGTGGACTATACTACGGAATGGTGCTTGCGCAAGATATAAACCAACAGACTGAA

ATGATCGATGATGAAATGGACGAGGTCGACGATGTAGACGACCGTAGTTCTAATTTGGATGTCGTTCGTAAGAAAAG
GTCAATTGCGAATTCTTACCACAGATCCGTGTCCGACCCTTCAGAATTATCTCTTAGGTCTTCAGCCGCAATCGTAAA
AGAGCTTCAGGACGCCGCGGAGGAGAGCTCAGTAAGACCTACACCGATGTCACGTATTTTTCTTATGAATAGAGAAA
CTTGGCCATATCTTTTCGTGGGACTGGTCGGTTGCTGCTTAAGTGGTATCGTCCCGCCCTTCTTTGCATTGGTTTATA
GTCAGATATTCAGCGTCTTCTCCGAACCGGTAGACAGACTTGGGAGCGACGCTCGTTTCTGGTCCCTTATGTTCTTA
GCATGTGGAGTGATCGATGCTATAGGTTTTTTCATTTCCGCCAACATGCTTGGGCTATGTGGAGAAACGCTGACGAA
AAAAATTAGGTTGATGGCTTTCACCAACTTATTGCGTCAAGATATAGCCTTCTATGACGATCAGAGACACTCAACGGG
GAAGCTTTGCACACGTTTTGCGACAGACGCGCCGAACGTCCGTTACGTCTTTACCCGTTTACCACTGGTAGTTGCGA
GCGTTGTAACTCTAGTCGGCGCTATTGCGATTGGGTTCCTGTTTGGGTGGCAACTAGCCTTAATCTTGCTAGCAATC
GTACCGCTAATTCTTGGCTCTGGTTATGTAGAAATGAGACTTCAGTTCGGGAAGCAGTTGAGGGAGACCGAGCTACT
AGAAGAAGCCGGGAGAACAGCGACCGAGGCCGTAGAGAATATACGTACAGTACAGTCACTTAATAAGCAGTCCGCT
TTTATCAGAGAATACTCACAACACTTGGAAACCCCTTTTAGGGAAAATATGCAGAGAGCACACATCTACGGAGCTGT
TTTCGCTTTCTCACAATCCTTAATCTTCTTCATGTATGCGTTAGCATTTTGGTTAGGTTCTTTATTTGTCGACAATGCG
GTGATGCAACCCATCAACGTTTACAGGGTGTTTTTCGCGATTGCTTTCTGCGGCCAAAGCGTGGGGCACATCAGTG
CCTTTATTCCGGACGTGGTTAAAGCACGTTTAGCCGCAAGTTTGGTCTTTCACCTAAGCGAGTATCCGACGGCCATA
GACTCATTGAGCGATCAAGGTAGTAGAATAACTATAAAGGGTGCTATCCAGTTAAAGAATGTTTTCTTTTCCTACCCG
ACAAGACGTAATACAAGAATATTAAGAGGCTTGACACTTAATGTCAAGGAGGGCGAAACGGTTGCACTTGTTGGGCA
CTCTGGCTGTGGGAAATCCACAGTTATGGGTCTGCTTGAGCGTTTTTACGACCCTAACAGAGGCAACATATACGTTG
ACGGAGAGAACATCAGAGACGTAAACATTAAGTGCTTGAGGTCCCAAATGTGTATTGTAAGCCAGGAACCCATACTA
TTTGATTGCACGATTGAGGAGAACATAATGTACGGACTGGACAGGGAAGTGAGCCATGAGGAGGTGGTCAACGCTG
CCAAACTGGCTAATATCCATAAATTTATCTTAAGCTTGCCCCTGGGGTATGAAACTAGGGTCGGTGAAAAAGGACC
CAGTTGAGCGGCGGACAAAAGCAGCGTATAGCGATAGCAAGGGCATTGATAAGAAATCCAAGCATTCTACTTCTAG
ACGAAGCAACCTCCGCTCTTGATACAGAATCTGAACAGGTCGTTCAAGAAGCTTTAGAAAACGCGCGTAAAGGTAG
GACTTGTCTTGTGATAGCCCATAGACTGAGCACAATTCAAAATTCAAATTTGATTGTTGTAATCAACGAGGGTAAGGT
TGCTGAGAAGGGCACCCATGCACAGTTGCTAGAGGCCAGCGGCATTTACAAAACACTGTGTGAGACGCAGACACTG
GTAGCTGCCAGTTATCCATACGATGTCCCTGATTATGCATGA

>*Pun*Pgp-9_codon_optimized
ATGTCCATTCGTAGCTCTCATAAGCGTAGAGATAACCTTTTGTCAGTCCCCTTATTGAGATCATTCAGAAAACGTCAG
AGCGAGGAACCGAAGGAGACCCCGCCGCCTAAGGCCTCCCTGAAACAACTGTTGCGTTATACCTCAACTTTTGACC
GTGCTTTGTTTGCGGTGGGCTCTCTAGTTGCCATTGGTACAGGCTGTGGGTTTCCGCTTCTGAGTATTCTAATGGGG
TCTATGTCCCAGTCCTTTGTCGACGCGCAGACCGCGTATTACGCTGGCTTGCGTCCGCATCTTAACACTACTATCGA
TAATGTAGAAGTCCCGCCTAGCTTGGAGAATTTCACCTGGGATGGCTTTAGTGACCAGGTGATTACATACTGTCTAG
ATTACGTTTGGATAGGTATTGCTATTTTATGCGCGGCAACGATTCAGGTGATGTGTTACCTTACTGCCTGTGAGAACA

TGAACCACAGAATGAGAAAGGAGTTCTTCAAGGCGGTTATCAGGCAAGATATCGGGTGGTTTGACGAAAATCAATCA
GGAACCCTGACTGCTAAGCTTTTCGACAACTTAGAAAGGGTGAAAGAGGGTACCGGTGATAAGATTGCTTTAATGAT
CCAGTTTATGAGTCAATTCTTCGCCGGCTTTATCATCGCATTTACATACGATTGGAGATTGACACTGATTATGATGAG
CCTGTCTCCTTTTATGGTAATTTGTGGGGCATTCATGGCAAAATTGATGGCAAGCGCGACTGCTAAGGAGGCAGAGA
ATTATGCCGTTGCCGGGGGAATCGCAGAGGAGGTCTTAACGTCAATACGTACAGTGGTGGCTTTCAACGGGCAGAA
GATTGAGTGCGACAGATATAACGAAGCTCTAAAAGGAGGAATGAGGGATGGGATATTAAAATCTTTGTACGTAGGTA
TAGGGCTTGGGCTTACTTTCTTCATTATATTCGGTTCCTACGCCCTAGCGTTCTGGGTTGGCACGGGATATGTGTAC
AATGACGTTCTAATTCCAGGTACCCTTCTAACTGTCTTTTTTGCGGTAATGATGGGCTCAATGGCGCTTGGGCAAGC
AGGCCCTCAATTCGCCGTCTTTGGGAGCGCTTTAGGCGCAGCCGGTGCGATTTTTGCAATAATTGATAGGGTGCCC
GAAATCGACACATATGACGAATCAGGGGAAAAGCCTGAACAGATGAAAGGCAAAATCGAACTTAGGAATATTGAGTT
CAGTTACCCGACGAGACCGGATATCAAAATACTAAACGGAATTTCTTTTTCCGTTAATCCTGGGGAAACAGTTGCGTT
GGTAGGCACTAGCGGCTGTGGCAAGTCTACCATAGTGTCTCTTTTGTTACGTTATTACAACCCGGAGTATGGAAACA
TCCTGATTGACGGCCATGAGATTTCTTCACTGAACTTGGCGTATCTGCGTAAGATGATCGGCGTTGTATCCCAGGAA
CCAGTTCTGTTTAATATGACGATTAAGGAGAACATCGAAATGGGCAACGAGGATGTTACCGAGGGAGAGATCTTGG
CCGCTTGCAGACGTGCTAACGCTACAAACTTCATTAACCAACTTCCGAATAAGTACGAAACGATAGTGGGTGACAGA
GGTACACAACTAAGTGGAGGGCAAAAACAAAGAATCGCTATAGCGCGTGCGCTAGTTAGGAACCCAAAAATACTTCT
ATTAGACGAAGCTACTTCCGCTCTAGACGCAGAGAGCGAATCAATAGTGCAGGAGGCGTTAGAGAAAGCAGCTCAG
GGACGTACCACAATCGTGATAGCGCATAGATTGTCTACTATCAAGAACGCGGACAAGATCATCGCAATGAAAGATGG
ACGTATCATTGAGATTGGAACGCACAACGAGCTTATTGCGGCCAATGGTTTTTATAGAGAGCTTGTGAATGCCCAGG
TATTCGCAGACGTTGATGAGAAACCCGCTCAGGAGAAAAGAACCACTGCCTATAGAAGCCCCAGTATTATAAGCAGA
AGGAGCAGGCTTAGCTCCACCATGTCCGAGAAAGATGCGCCTTTGTCTCCTAAGTTCACCAGTAGAATAGAATCTGA
GACTGGGGTCGACCCCACTGACCAGCAGGACATAAAGAAAGAAACGGAGAGGTTGCGTAAAGAAATGGAGGAGGA
AGGCGTGACAGAGTCTAATTTGATAGAAATACTTCAGTACGCTAGACCTGAGTGGATATATATAATATTAGGCTTGTT
CGCATGCCTTATACAAGGTAGTGTATATCCTGTCTTTAGTTTACTATTTAACGAGATTCTGGCGATCTTCGCGAAACC
TAGGGATCAGATGCTAAGTAATGGACACTTTTACTCCCTGATGTTCTTGGTGGTGGGTGCGGTATCTGCGACTACAC
TACTAATTCAGGCCTTTTTCTTTGGTATGTCCGCCGAGCGTCTTACGAGAAGGTTAAGGTCACGTATATTTCAGAACG
TACTTAGGATGGATATTGCGTACTTCGACATGCCTAACCATTCAAGCGGCAAGATTTCCACGAGGCTGGCCACGGA
CACACCAAATGTGAAGTCTGCCATAGACTATAGACTGGGCTCAGTTTTAAGCAGCCTGGTTTCAGTAGGTTTCGGGA
TAGGCATTGCTTTTTATTTTGGTTGGCAGATGGCACTTCTTGTTATCGCTATATTCCCGCTTGCAGGTGTCGGCCAG
GCTTTCCACTTAAAGTATATCGAGGGCAGGCACAATCAAGATGCTAAGGAGCTTGCCAGCTCTGGTAAGGTCGCTTT
GGAAGCGATTGAAAGTATCAGGACGGTTCATGCCCTAACCCTAGAGCGTAAATTCTATGAGAAATTCTGCCATTTCT
TAGAGCGTCCTCATGAAACATCAACGCGTAAGGCGATAGCCCAGGGCATGGCTTACGGTTTTGCGAATTCTATATTC
TACTTCTTGTACGCCTCTGCTTTCAGGTTTGGGTTATTTTTAATTTTAGAGGAGATCAGCAGTCCAATAAATGTAATGA
AGGTGTTATTTGCCATATCTTTTACAGCCGGTACGTTAGGGTTCGCGTCAGCGTACTTTCCAGAGTATGCGAAAGCA

AAATTCGCTGCCGCAATAATTTTTAAAATGTTGAAGGAGGAACCTAAGATCGACTCTATGAAAACTGATGGCGAGAA
GCCGGAGATTTCCGGGTCCGTCGATTTTTCCAAGATCTATTTCGCATATCCCGAGCGTCCTGAAGTCGGCGTCCTAA
AGGGCCTGGATTTACATGTAGACGCCGGGCAGACCCTGGCAATAGTAGGACCGAGCGGGTGTGGAAAGAGTACTG
TCGTCAGCCTACTAGAACGTTTTTACGATCCGATTGACGGTACTGTCCGTGTCGACGGGAACGACATAAGACTTATC
AATCCGACATATTTGAGATCACAACTGGCTCTTGTCTCACAAGAGCCCATCTTGTTCGATTGCAGCATCAGGGAAAA
TATCATCTACGGCCTTCAGACGGAGGAATTTTCCGATGAAGACATCGTGAATGTGGCGAGGTTGGCAAATATCGACA
AATTTATTAAAGAGCTGCCAGATGGTTATGAGACCAGAGTCGGGGAAAAGGGTACCCAACTAAGCGGGGGACAGAA
GCAACGTATAGCGATAGCGCGTGCGTTAATTAGGCAACCCAAAATACTATTGCTTGATGAAGCTACAAGTGCTCTAG
ATACAGAGTCAGAGAAAGTGGTACAGGAGGCACTAGATAGGGCCGGGAAAGGTAGGACGTGTATAATAATAGCTCA
CAGGCTGTCAACAGTAGTGAATGCAAATTGCATTGCCGTGGTGAAGAACGGCATTGTTCTGGAAAAAGGACCCAC
GCAGAACTAATGGATAAGCGTGGTGCCTATTACTCACTAACACAAAAACAGAATTTGAAGGAGACTGACGACGATGA
CGATGATTTTGACGCAAGAATGTACCCATACGACGTTCCGGACTATGCTTGA

e) Primers for validation of constructs by sequencing

Gene	Forward Primers	Sequence (5'-3')	Reverse Primers	Sequence (5'-3')
*Pun*Pgp-2 codon optimized	M13-24F-BLUE	GTAAAACGACGGCCAGTGAGCGCG	M13-24R-BLUE	AACAGCTATGACCATGATTACGCC
	Pgp-2_fw_1	TGATGCGTTCAAGTCAGGATAATG		
	Pgp-2_fw_2	AACATGCTTGGGCTATGTGGAG		
**Gene**	**Forward Primers**	**Sequence (5'-3')**	**Reverse Primers**	**Sequence (5'-3')**
*Pun*Pgp-9 codon optimized	M13-24F-BLUE	GTAAAACGACGGCCAGTGAGCGCG	M13-24R-BLUE	AACAGCTATGACCATGATTACGCC
	Pgp-9_fw_1	ATGTCCATTCGTAGCTCTCATAAG		
	Pgp-9_fw_2	AACGAGATTCTGGCGATCTTCG		

f) **Primers for RT-PCR for validation of transgene expression in yeast**

Gene	Direction	Sequence (5'-3')	Tm (°C)	Fragment Size (bp)
*Pun*Pgp-2 Codon optimized	Forward	GCATAATCAGGGACATCGTATGG	63.9	962
	Reverse	GCGGTGATGCAACCCATCAAC		
*Pun*Pgp-9 Codon optimized	Forward	TGGCCACGGACACACCAAATGTG or TAGCCCAGGGCATGGCTTACGGTTT (used for RT-PCR in Supplemental Fig. 3)		1441 or 1085
	Reverse	GCATAGTCCGGAACGTCGTATGG		
lacZ	Forward	CTTTTGACACCAGACCAACTGG	63.1	1141
	Reverse	TGTTCCGTCATAGCGATAACG		

Supplementary Table S2. Primers and sequences Sequences of a) full length open P-glycoprotein reading frame amplification (RT-PCR) primers for all *Parascaris univalens* Pgp, b) additional primers used to obtain sequences missing in both transcriptomes, verification primers, RACE PCR and spliced leader PCR) c) sequencing primers for all *Parascaris* Pgp, made by primer walking, d) Codon optimized constructs of *Pun*Pgp-2 and *Pun*Pgp-9 for expression in *Saccharomyces cerevisiae*, e) primers for validating correctness of codon optimized yeast expression constructs e) primers for RT-PCR for validating transgene expression at the transcription level. *Pun*: *Parascaris univalens*; Pgp: P-glycoprotein

Supplementary Table S3

Gene Name in Maximum Likely Hood Tree	Annotated Name (if applicable)	Organism	Accession Number Genbank	WormBase ID	Contig
***Pun*Pgp-11,1**	***Peq*Pgp-11**	*Parascaris univalens*	AGL08022.1		
***Pun*Pgp-16.1**	***Peq*Pgp16**	*Parascaris univalens*	AGL08023.1		
***Pun*Pgp-2**		*Parascaris univalens*	MT001899		
***Pun*Pgp-3**		*Parascaris univalens*	MT001900		
***Pun*Pgp-9**		*Parascaris univalens*	MT001901		
***Pun*Pgp-10**		*Parascaris univalens*	MT001902		
***Pun*Pgp-11.2**		*Parascaris univalens*	MT001904		
***Pun*Pgp-12**		*Parascaris univalens*	MT001905		
***Pun*Ppg-16.2**		*Parascaris univalens*	MT001907		
***Pun*Pgp-18A**		*Parascaris univalens*	MT001908		
***Pun*Pgp-18B**		*Parascaris univalens*	MT001909		
***Cbr*Pgp-16**	Hypothetical protein CBG12969	*Caenorhabditis briggsae*	CAP31851.1	BP:CBP17602	
***Cel*Pgp-1**		*Caenorhabditis elegans*	NP_502413	WP:CE11932	
***Cel*Pgp-2**		*Caenorhabditis elegans*	NP_491707	WP:CE41207	
***Cel*Pgp-3**		*Caenorhabditis elegans*	NP_509901	WP:CE03818	
***Cel*Pgp-4**		*Caenorhabditis elegans*	NP_001257143	WP:CE03308	
***Cel*Pgp-5**		*Caenorhabditis elegans*	NP_001257116	WP:CE43003	
***Cel*Pgp-6**		*Caenorhabditis elegans*	NP_001041287	WP:CE40818	

***Cel*Pgp-7**		*Caenorhabditis elegans*	NP_509812	WP:CE36668	
***Cel*Pgp-8**		*Caenorhabditis elegans*	NP_509811	WP:CE31624	
***Cel*Pgp-9**		*Caenorhabditis elegans*	NP_507487	WP:CE15714	
***Cel*Pgp-10**		*Caenorhabditis elegans*	NP_509205	WP:CE40807	
***Cel*Pgp-11**		*Caenorhabditis elegans*	NP_495674	WP:CE34788	
***Cel*Pgp-12**		*Caenorhabditis elegans*	NP_510126	WP:CE03260	
***Cel*Pgp-13**		*Caenorhabditis elegans*	NP_510127	WP:CE40253	
***Cel*Pgp-14**		*Caenorhabditis elegans*	NP_510128	WP:CE0262	
***Ppa*Pgp-1**		*Pristionchus pacificus*		PP:PP30697	
***Ppa*Pgp-9**		*Pristionchus pacificus*		PP:PP38391	
***Asu*Pgp-2**	Multidrug resistance protein 1	*Ascaris suum*	ADY40620		
***Asu*Pgp-11.2**	Multidrug resistance protein 3	*Ascaris suum*	ADY40644		
***AsuPgp*-16.2**	Multidrug resistance protein 3	*Ascaris suum*	ADY40573		
***Asu*Pgp-2_hp**		*Ascaris suum*			AgR011_g279_t03
***Asu*Pgp-3_hp**		*Ascaris suum*			AgR049_g026_t04
***Asu*Pgp-9_hp**		*Ascaris suum*			AgB03_g189_t01
***Asu*Pgp-10_hp**		*Ascaris suum*			AgR054_g025_t01
***Asu*Pgp-11.1_hp**		*Ascaris suum*			AgB01_g256_t02
***Asu*Pgp-12_hp**		*Ascaris suum*			AgR009X_g036_t04
***Asu*Pgp-16.1_hp**		*Ascaris suum*			AgR016_g207_t01
***Asu*Pgp-18A_hp**		*Ascaris suum*			AgR067_g008_t02
***Asu*Pgp-18B_hp**		*Ascaris suum*			AgR067_g008_t02

***Hco*Pgp-2**		*Haemonchus contortus*	AAC38987		
***Hco*Pgp-3**		*Haemonchus contortus*	AFX93749.1		
***Hco*Pgp-13**		*Haemonchus contortus*	ARE67135.1		
***Hco*Pgp-16**		*Haemonchus contortus*	AFX93754.1		
**HcoPgp-11.2**		*Haemonchus contortus*	CDJ81808.1		
***Hco*Pgp-11.1**		*Haemonchus contortus*	CDJ84338.1		
***Hco*Pgp-10**	unnamed protein product	*Haemonchus contortus*	CDJ83820.1		
***Hco*Pgp-9.1**		*Haemonchus contortus*	JX430397		
***Con*Pgp-2**		*Cooperia oncophora*	AGJ71178		
***Con*Pgp-3**		*Cooperia oncophora*	AGJ71177		
***OvoPgp-3 (*10280)**		*Onchocerca volvulus*		OV:OVP11414	
***Ovo*Pgp-10**		*Onchocerca volvulus*		OV:OVP13603	
***Ovo*Pgp-11**		*Onchocerca volvulus*		OV:OVP14503	
***Dim*Pgp-3**		*Dirofilaria immitis*	ALI16766.1		
***Dim*Pgp-11**		*Dirofilaria immitis*	ALI16773.1		
***Dim*Pgp-10**		*Dirofilaria immitis*	ALI16764.1		
***Bma*PGP-10**		*Brugia malayi*		BM:BM29899	
***Bma*Pgp-21**		*Brugia malayi*	XP_001900095		
***Bma*Pgp-10a**		*Brugia malayi*	XP_001897744		
***Bma*Pgp11**		*Brugia malayi*	CRZ23051.1		
***Bma*Pgp-3**	Bm11309	*Brugia malayi*		BM:BM42091	
***Bma*Pgp-16**	Bm2594, isoform a	*Brugia malayi*	CDP97625.1		
***Nam*Pgp-2**	ABC transporter, ATP-binding protein	*Necator americanus*	XP_013305476.1		
***Nam*Pgp-13**	ABC transporter, ATP-binding protein	*Necator americanus*	XP_013298646.1		

***Nam*Pgp-10**	ABC transporter, ATP-binding protein	*Necator americanus*	XP_013299120.1		
***Nam*Pgp-14**	hypothetical protein NECAME_00050	*Necator americanus*	XP_013309418.1		
***Celo*Pgp-9**		*Cylicocyclus elongatus*	AJM87336.1		
***Tci*Pgp-13**		*Teladorsagia circumcincta*	PIO77339.1		
***Tci*Pgp-9**		*Teladorsagia circumcincta*	PIO69295.1		
***Sma*MRP1_1 (1,2,3)**		*Schistosoma mansoni*	XP_018648325.1		
***Sma*MRP1_2 (1,2,3)**		*Schistosoma mansoni*	XP_018651783.1		
TcaPgp-2	Multidrug resistance protein 1A	*Toxocara canis*	KHN80157.1		
***Tca*Pgp-3a**		*Toxocara canis*	KHN78383.1		
***Tca*Pgp-12**	Multidrug resistance protein pgp-3	*Toxocara canis*	KHN87068.1		
***Tca*Pgp-11.1**	Multidrug resistance protein pgp-1	*Toxocara canis*	KHN73709.1		
***Tca*Pgp-11.2**	Multidrug resistance protein pgp-3	*Toxocara canis*	KHN89031.1		
TsuPgp-2	hypothetical protein M514_09538	*Trichuris suis*	KFD70158.1		
**Outgroup (collapsed)**	**-**				
***Dme*PgpA**		*Drosophila melanogaster*	AAA16186		
***Dme*PgpB**		*Drosophila melanogaster*	AAA28679		
***Dme*PgpC**		*Drosophila melanogaster*	AAA28680		
***Mca*Pgp-L**		*Mytilus californianus*	ABS83556		
***Mga*Pgp-L**		*Mytilus galloprovincialis*	CAX46411		
***Mmu*ABCB-1b**		*Mus musculus*	NP_035205		
***Mmu*Pgp**		*Mus musculus*	AAA39514		

***Phu*MRP-1**		*Pediculus humanus corporis*	XP_002425149		
***Phu*MRP-2**		*Pediculus humanus corporis*	XP_002432260		
***Phu*MRP-3**		*Pediculus humanus corporis*	XP_002426586		
***Phu*MRP-4**		*Pediculus humanus corporis*	XP_002425021		
***Hsa*ABCB1**		*Homo sapiens*	NP_000918.2		

Supplementary Table 3. NCBI Accession numbers, WormBase IDs and contigs (*Ascaris suum* transcriptome, GEO: GSE38470) of protein sequences used in the maximum likelihood tree. Annotated gene names were changed according to the result of the phylogenetic analysis in the tree and original gene names are listed.
